# Exploring the mechanism of Danggui Sini Decoction in the treatment of myocardial infarction: A systematic review, network pharmacology, and molecular docking

**DOI:** 10.1097/MD.0000000000040073

**Published:** 2024-10-18

**Authors:** Zhenzhen Li, Shuang Liu, Rui Zhang, Bing Li

**Affiliations:** a Guizhou University Medical College, Guiyang, Guizhou, PR China.

**Keywords:** Danggui Sini Decoction, molecular docking, myocardial infarction, network pharmacology

## Abstract

Myocardial infarction (MI) is one of the leading causes of death worldwide because of its high morbidity and mortality. Traditional Chinese Medicine compounds play a crucial role in preventing cardiovascular diseases. Danggui Sini Decoction (DSD) is widely used clinically for cardiovascular diseases. However, the mechanism, main components, and main targets of DSD in treating MI are still unclear. In this study, we utilized network pharmacology and molecular docking for exploration. MI-related genes were examined using the Genecards database, and the active ingredients of DSD were screened based on System Pharmacology Database and Analysis Platform of Traditional Chinese Medicine by oral bioavailability ≥ 30% and drug-likeness ≥ 0.18. The protein–protein interaction network diagram was generated using the STRING database. The DAVID web platform was used to carry out gene ontology and Kyoto encyclopedia of gene and genome signaling pathway analysis. DSD’s screening study revealed 120 primary active ingredients and 561 putative active target genes. The main therapeutic targets were TP53, EGFR, AKT1, IL6, TNF, STAT3, IL1B, CTNNB1, SRC, MYC, JUN, and INS. Gene ontology and Kyoto encyclopedia of gene and genome analyses revealed that DSD treatment of MI mainly involves the positive regulation of the ERK1 and ERK2 cascades, positive regulation of cell proliferation, inflammatory responses, aging, and the MAPK cascade, along with other biological processes. The molecular docking results indicate that DSD drugs may interact with AKT1, EGFR, TP53, and TNF through formononetin, isorhamnetin, β-Sitosterol, and kaempferol, potentially contributing to the treatment of MI. By utilizing a multi-component, multi-pathway, and multi-target mode of action, DSD may have the potential to prevent MI.

## 1. Introduction

Myocardial infarction (MI) is one of the leading causes of death worldwide due to its high morbidity and mortality.^[1]^ The high mortality rate of MI is primarily attributed to the complex pathogenesis and influencing factors, including inadequate blood and oxygen supply to the heart, increased cardiac oxygen consumption, various triggering factors, and severe complications.^[2,3]^ MI is caused by the rupture of atherosclerotic plaque and thrombosis, resulting in the occlusion of coronary arteries. This results in reduced blood supply and necrosis of cardiomyocytes. The heart primarily relies on oxidative metabolism for energy synthesis and is therefore highly sensitive to changes in intracellular oxygen concentration.^[4,5]^ Decreased oxygen levels lead to myocardial energy starvation and cardiomyocyte dysfunction.^[6,7]^ Due to the heart’s limited capacity for regeneration, the infarct site undergoes a remodeling process.^[8,9]^ The current clinical program for managing MI primarily includes percutaneous coronary interventions, coronary artery bypass grafting, antiplatelet drugs, and thrombolytic therapy. Despite the significant therapeutic benefits associated with these interventions, they carry surgical risks and may lead to complications such as bleeding, ischemia–reperfusion injury, and restenosis of the coronary arteries. Additionally, these treatments often come with a significant financial burden for patients. Traditional Chinese Medicine (TCM) emerges as a promising alternative, showcasing its unique strengths in this field.

Recent studies have shown that TCM compounds play a significant role in preventing cardiovascular diseases. Danggui Sini Decoction (DSD), for example, can regulate blood pressure and improve blood flow characteristics.^[10]^ DSD originated from the “Treatise on Typhoid Fever” by Zhongjing Zhang, a renowned expert in typhoid. The composition of DSD includes: *Angelica sinensis* (Danggui, DG), *Ramulus Cinnamomi* (Guizhi, GZ), *peony* (Shaoyao, SY), *asarum* (Xixin, XX), *Glycyrrhiza uralensis* (Gancao, GC), *tongcao* (TC), and *jujube* (Dazao, DZ). DSD is mainly used for the treatment of syndromes associated with typhoid fever. DSD has been widely used in clinics for cardiovascular diseases, peripheral blood circulation disorders, and thrombotic diseases. Although the formula for DSD is rigorous, the drug combination is exquisite, and the therapeutic efficacy is remarkable, the material basis of the pharmacodynamic effect has not been completely elucidated. Therefore, our study investigates the potential mechanism of DSD in the treatment of MI with the aim of providing valuable references for its clinical application.

Network pharmacology is an emerging discipline based on the development of systems biology and the improvement of network databases. It constructs the network signaling relationship between “TCM-ingredients-targets-diseases” through network analysis and has the characteristics of “multi-disciplinary.” It can be applied to the new model of complex network relationships between multiple targets and multiple diseases.^[11]^ TCM has the synergistic effects of multiple components, pathways, and targets, emphasizing the holistic impact of drugs on the body network. Network pharmacology aligns with the holistic approach of Chinese medicine,^[12]^ giving it a distinct advantage in the study of Chinese medicine. In this study, we utilized network pharmacology to investigate the potential mechanism of clinical application of DSD in the treatment of MI. This exploration may offer a theoretical foundation for the clinical use of DSD.

## 2. Methods and materials

### 2.1. Flowchart of the method

**Figure 1.**
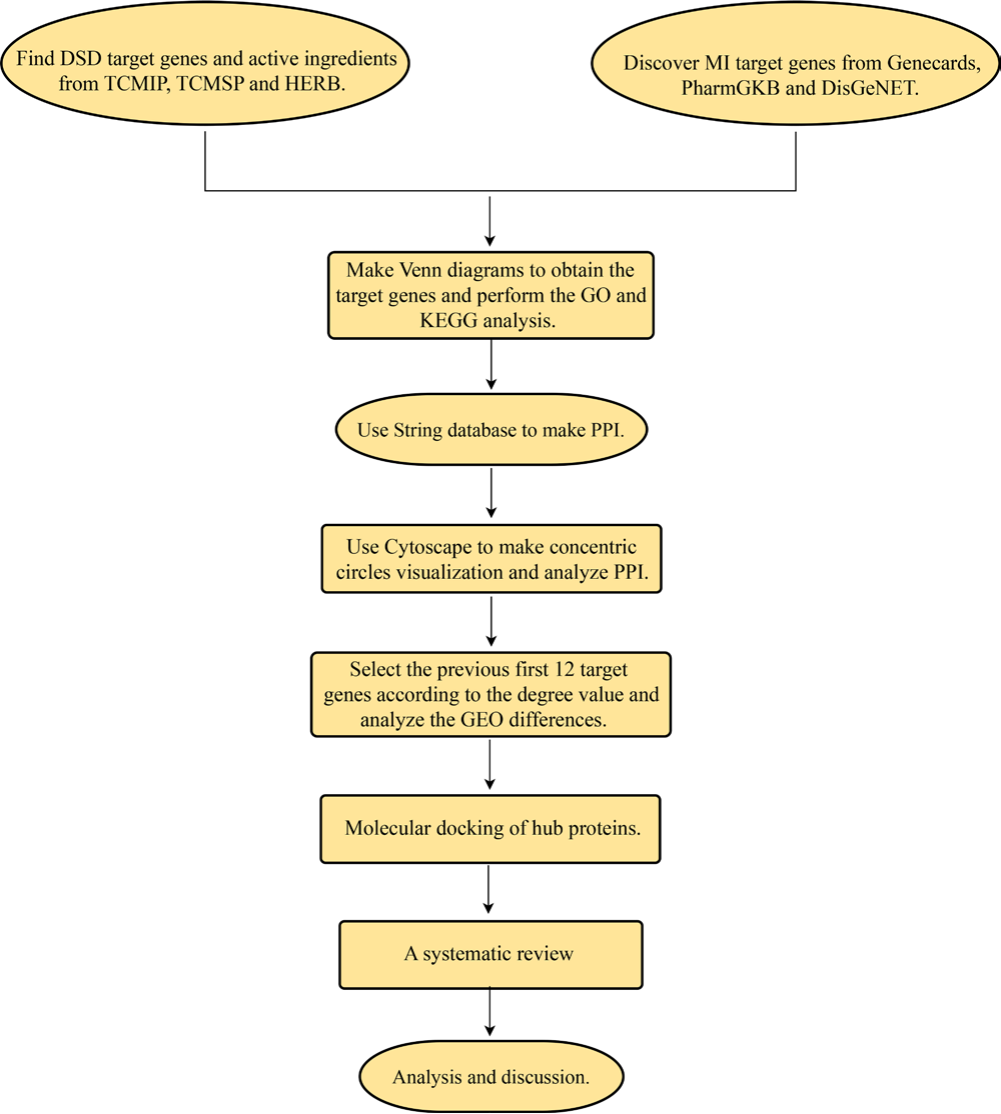
Flow chart of this study.

In order to identify the intersecting target genes for treating MI in patients with DSD, the target genes for DSD from the Integrated Pharmacology Research Platform of Traditional Chinese Medicine (TCMIP), System Pharmacology Database and Analysis Platform of Traditional Chinese Medicine (TCMSP), and a high-throughput experiment and reference-guided database of traditional Chinese medicine (HERB) databases were compared with the target genes for MI from the Genecards, DisGeNET, and Pharmgkb databases using the Venn diagram tool. From there, the identified target genes at the intersection were subjected to enrichment analysis to generate gene ontology (GO) and Kyoto encyclopedia of genes and genomes (KEGG) analyses. We imported the intersection target genes into the STRING database for protein-protein interaction (PPI) networks and used Cytoscape software for PPI visualization and analysis. We then used Cytoscape to extract the key protein topology parameters from the protein interaction network of DSD in the treatment of MI, which led to the identification of 12 key target genes. Finally, differential expression analysis was conducted to confirm the statistical significance of the expression of key target genes in the Sham and MI samples. Molecular docking was also performed to validate the binding ability of the active ingredients to the key targets. Figure 1 displays the flowchart outlining the methodology of this study.

### 2.2. Screening of components from DSD

To acquire the chemical components of each herbal ingredient in DSD, and to identify the relevant chemical components and their corresponding target information, utilize the network information platforms provided by the TCMIP v2.0 (http://www.tcmip.cn/), the TCMSP (https://old.tcmsp-e.com/tcmsp.php), and the HERB (http://herb.ac.cn). The names of all the TCMs in DSD were retrieved using TCMIP, and the relevant chemical components and target information were collected directly. To find the matching chemical components, we searched the names of all the TCMs in DSD using TCMSP. Oral bioavailability (OB) ≥ 30% and drug-likeness (DL) ≥ 0.18 were utilized as screening thresholds with the TCMSP to identify the eligible chemical components and their respective target information.

### 2.3. Acquisition of disease targets

The term “myocardial infarction” was searched in the DisGeNET (https://www.disgenet.org/search), Pharmgkb (https://www.pharmgkb.org/), and Genecards (https://www.genecards.org/) databases to find MI-related gene targets.

### 2.4. Predicting therapeutic targets for DSD

Using Venny 2.1.0 (https://bioinfogp.cnb.csic.es/tools/venny/index.html) to identify the overlapping targets of DSD and MI-related targets, we can obtain potential pharmacological targets for DSD to treat MI.

### 2.5. PPI network of target protein interaction

We utilized the STRING database (https://cn.string-db.org/) to retrieve information on prediction and experimental protein interactions. Select the species “Homo sapiens” from the “Organisms” dropdown menu after choosing “Multiple proteins” in the search area of the STRING database. To obtain the PPI network, click “SEARCH” again, export the “.tsv” file, and visualize it using Cytoscape 3.10.1. We selected the top 12 target genes as key genes based on their degree value after calculating the topology parameters of betweenness centrality, closeness centrality (CC), and degree centrality using the Cytoscape plug-in CytoNCA.

### 2.6. GO and KEGG pathway enrichment analysis

The gene function analysis tool DAVID (https://david.ncifcrf.gov/summary.jsp) was utilized to conduct GO enrichment and KEGG signaling pathway analysis of potential therapeutic target genes for DSD treatment of MI obtained from the screening.

### 2.7. Analysis of differences

Utilizing the GEO database (https://www.ncbi.nlm.nih.gov/geo/) to access sample data. In 16 samples of GSE27962 (expression data of Sham and post-MI myocardium from swine), data related to the differential expression between the control group and the MI group for 12 key targets (selected based on degree value as the top 12 target genes) were collected. Statistical analyses were performed using SPSS 27.0 software (IBM, New York, NY). The data was analyzed for statistical differences using the *t* test. We calculated the significance of each treatment as a *P*-value, and *P* < .05 was considered statistically significant. We used GraphPad Prism. 9.1.0 for visualization.

### 2.8. Molecular docking methods

To further identify the critical genes, we filtered the top 5 core target genes in the core PPI network based on their degree values. We screened and confirmed the primary active ingredients associated with the “active ingredients-target gene” network using molecular docking.

The 3 crucial processes of molecular docking are ligand preparation, ligand-protein docking, and macromolecule preparation. (1) TCMSP provided the accurate 2D structures of the active ingredients required for ligand synthesis (in mol2 format). (2) The RCSB Protein Database (https://www.rcsb.org) provided the 3D protein structures. We used PyMOL 2.3.1 software to remove water molecules and invalid small-molecule ligands. We then conducted molecular docking using an online molecular docking website (https://cadd.labshare.cn/cb-dock2/php/blinddock.php) to ascertain the binding affinity and orientation of the active ingredients of the DSD drug with the target protein. (3) PyMOL 2.3.1 software visualized the results of successful molecular docking.

## 3. Results

### 3.1. Screening of active ingredients in DSD

We obtained a total of 307 chemical components and 5598 corresponding targets of DSD from the TCMIP, TCMSP and HERB databases. We integrated and listed the chemical components selected from TCMIP and TCMSP, along with the corresponding target information, based on the source of these active ingredients (Table S1, Supplemental Digital Content, http://links.lww.com/MD/N757). Of these, 29 are from *A sinensis*, 10 from *R Cinnamomi*, 39 from peony, 13 from asarum, 140 from *G uralensis*, 2 from tongcao, and 61 from jujube. The other 13 compounds, including quercetin, cetylic acid, hexadecanoic acid, palmitic acid, myristic acid, sitosterol, β-sitosterol, catechin, formononetin, stigmasterol, carvacrol, (+)-catechin, rutin, rutoside, vitamin P, phenol, sitosterol, and mairin, were also found in several other TCM, respectively. Meanwhile, we collected the constituent compounds of DSD in the HERB database (Table S2, Supplemental Digital Content, http://links.lww.com/MD/N757).

### 3.2. Obtaining information on the common targets of the active ingredients of DSD and MI

**Figure 2.**
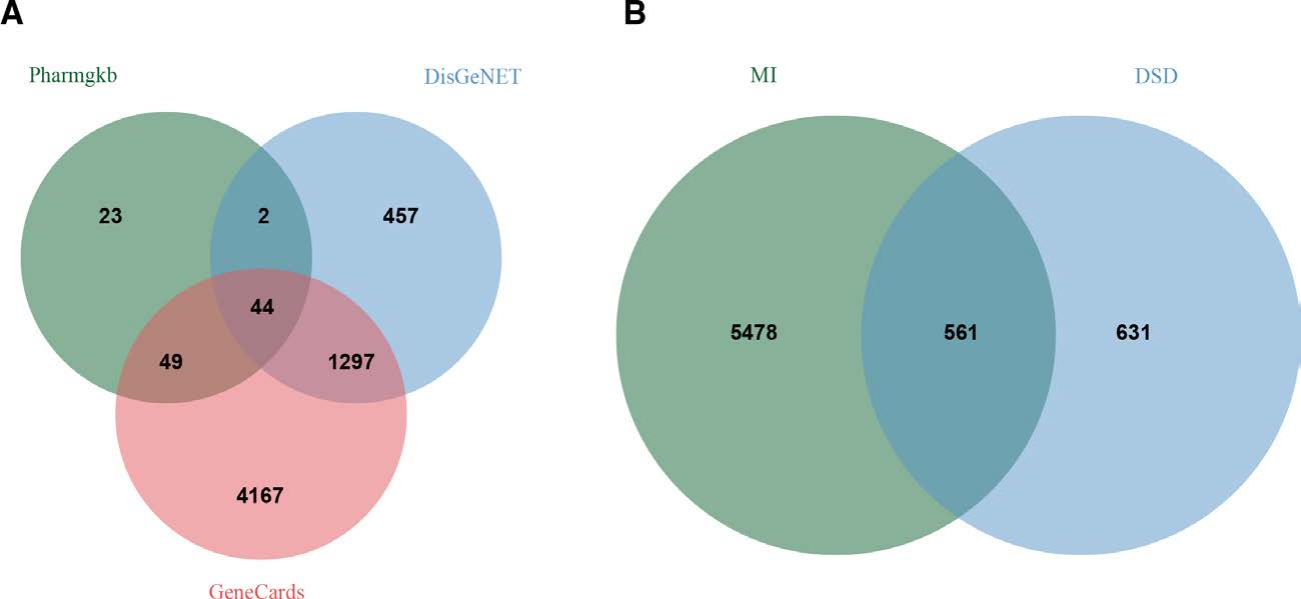
Overlapped genes between targets of DSD and MI-related genes from the GeneCards, DisGeNET, and Pharmgkb databases. (A) MI-related targets. (B) Overlapped genes. DSD = Danggui Sini Decoction, MI = myocardial infarction, Pharmgkb = pharmacogenetics and pharmacogenomics knowledge base.

We obtained 5598 drug-gene interactions related to DSD from the TCMIP, TCMSP, and HERB databases. Additionally, we obtained 6039 MI-related genes from Genecards, DisGeNET, and Pharmgkb databases (Fig. 2A). By intersecting disease and drug-related genes, we identified 561 genes (Fig. 2B). It is highly likely that DSD may exert its therapeutic effects on MI through these targets.

### 3.3. Construction of PPI network

**Figure 3.**
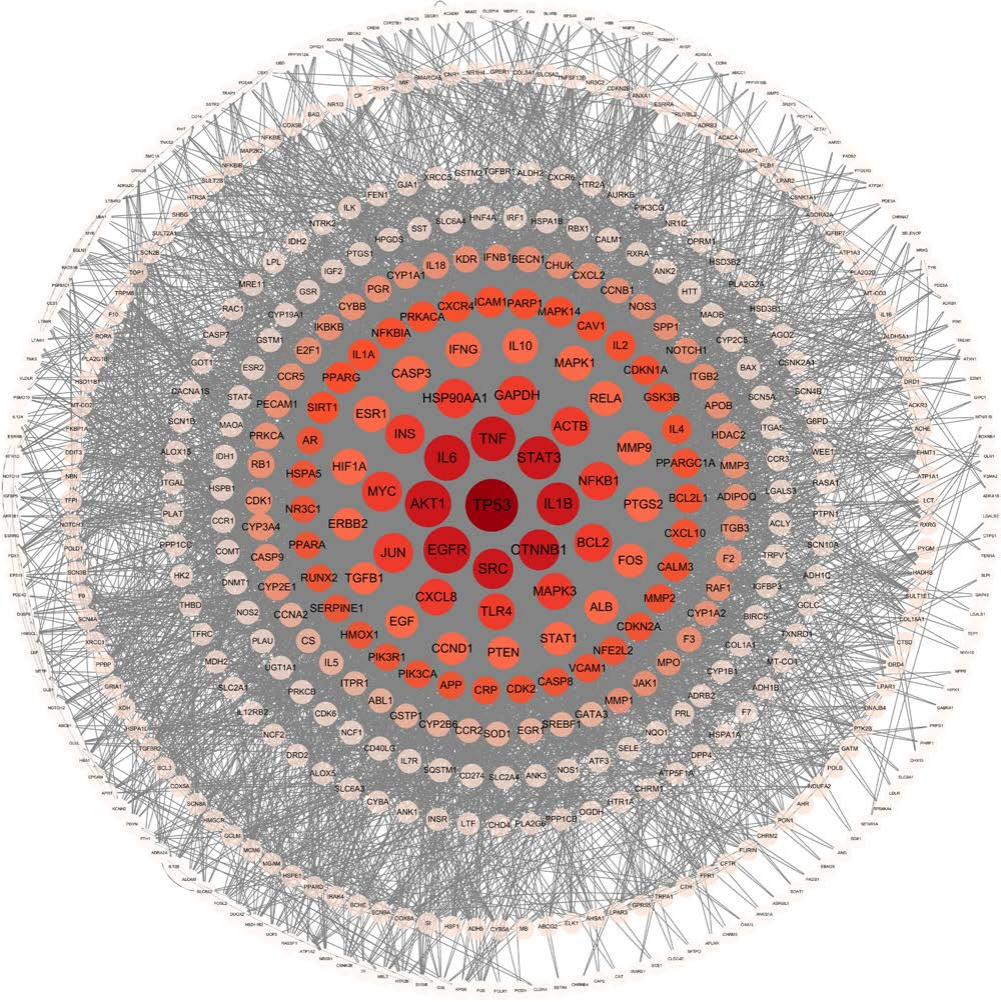
PPI network of the 561 intersection targets of DSD and MI. Deep red represents the core targets, light red represents the sub-core targets, and pink represents the intersection targets other than the core and sub-core targets. The darker the color, the higher the degree value. DSD = Danggui Sini Decoction, MI = myocardial infarction, PPI = protein–protein interaction.

We loaded the 561 intersection targets mentioned above into the STRING database and ran PPI analysis. There are 508 nodes and 7984 edges in the network overall (Fig. S1, Supplemental Digital Content, http://links.lww.com/MD/N757). After visual processing with Cytoscape software, the PPI network graph is obtained. A larger node indicates a higher degree value and more related genes; the lines connecting nodes represent interactions between genes (Fig. 3). Based on an analysis of the PPI network’s topological features, the average betweenness centrality was 0.003968223, the average CC was 0.341618558, and the average degree centrality was 31.43307087. Table 1 presents the top 12 targets along with their corresponding topological parameters, sorted from highest to lowest by degree value. The targets listed are TP53, EGFR, AKT1, IL6, TNF, STAT3, IL1B, CTNNB1, SRC, MYC, JUN, and INS, which are likely to be the primary focus of DSD in the treatment of MI.

**Table 1:** Key protein topological parameters of the protein interaction network in DSD treatment of myocardial infarction.

No.	Gene symbol	Degree	Betweenness centrality	Closeness centrality
1	TP53	258	0.10484960323567925	0.5014836795252225
2	EGFR	214	0.0736244883386346	0.49754661432777236
3	AKT1	210	0.04628911636121703	0.49463414634146347
4	IL6	204	0.03189372374863607	0.4778510838831292
5	TNF	196	0.043334297360764544	0.4889103182256509
6	STAT3	184	0.020123997990470148	0.4690101757631822
7	IL1B	180	0.02812176866668172	0.4668508287292818
8	CTNNB1	166	0.029105747338659116	0.47118959107806685
9	SRC	162	0.029238452111231803	0.47075208913649025
10	MYC	158	0.020617336612309432	0.4563456345634564
11	JUN	158	0.015276054065491077	0.4725069897483691
12	INS	154	0.0650473579740451	0.4725069897483691

### 3.4. GO and KEGG enrichment analysis

**Figure 4.**
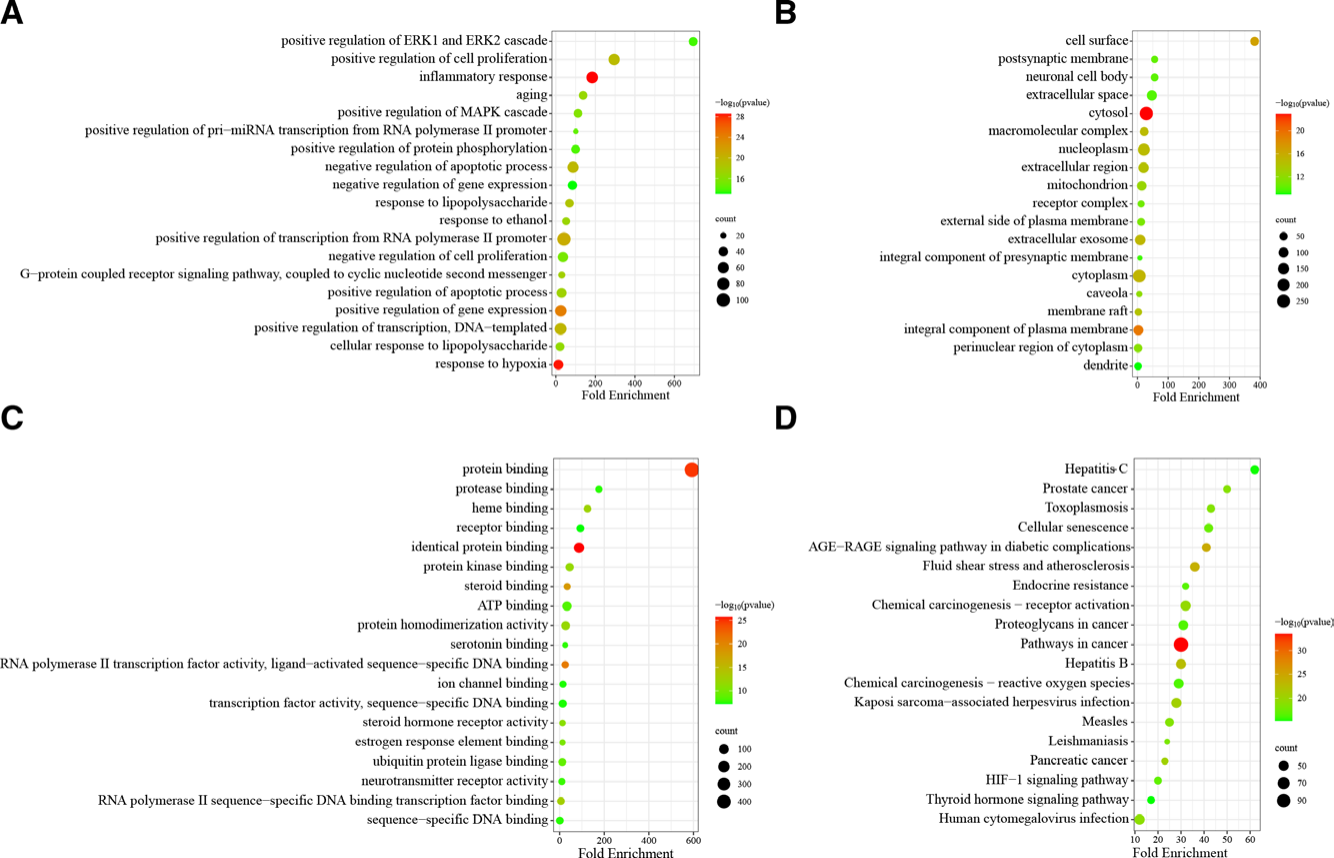
GO and KEGG enrichment analysis of the target proteins. (A) BP bubble chart; (B) CC bubble chart; (C) MF bubble chart; (D) KEGG bubble chart. BP = biological process, CC = cell component, GO = gene ontology, KEGG = Kyoto encyclopedia of gene and genome, MF = molecular function.

The DAVID system was used to import the intersecting genes of the active constituents of DSD and MI for GO and KEGG enrichment analysis. We collected information on biological processes (BP), CC, molecular function, and KEGG pathways. As can be seen from the analysis of BP, analysis, the BP mainly involved in the key targets includes positive regulation of ERK1 and ERK2 cascades, positive regulation of cell proliferation, inflammatory response, aging, and positive regulation of MAPK cascade (Fig. 4A). According to the analysis of CC, the key targets mainly acted on the cell surface, postsynaptic membrane, neuronal cell body, extracellular space, and cytosol (Fig. 4B). Molecular function analysis shows that the key targets are predominantly involved in protein binding, protease binding, heme binding, receptor binding, and identical protein binding (Fig. 4C). Furthermore, we obtained the top 20 pathways through KEGG, which included Hepatitis C, prostate cancer, toxoplasmosis, cellular senescence, and the AGE-RAGE signaling pathway in diabetic complications (Fig. 4D). It reflects the network mechanism of multi-ingredients, multi-pathway, and multi-target. We have identified 2 pathways closely related to MI: lipid and atherosclerosis, and fluid shear stress and atherosclerosis. Within these 2 pathways, we have identified 9 key intersection genes (TP53, AKT1, IL6, TNF, IL1B, SRC, JUN, CTNNB1, and STAT3) associated with MI. This indirectly suggests that drugs for DSD likely exert their therapeutic effects on MI through these targets (Figs. S2, Supplemental Digital Content, http://links.lww.com/MD/N757 and S3, Supplemental Digital Content, http://links.lww.com/MD/N757).

### 3.5. Analysis of differences

**Figure 5.**
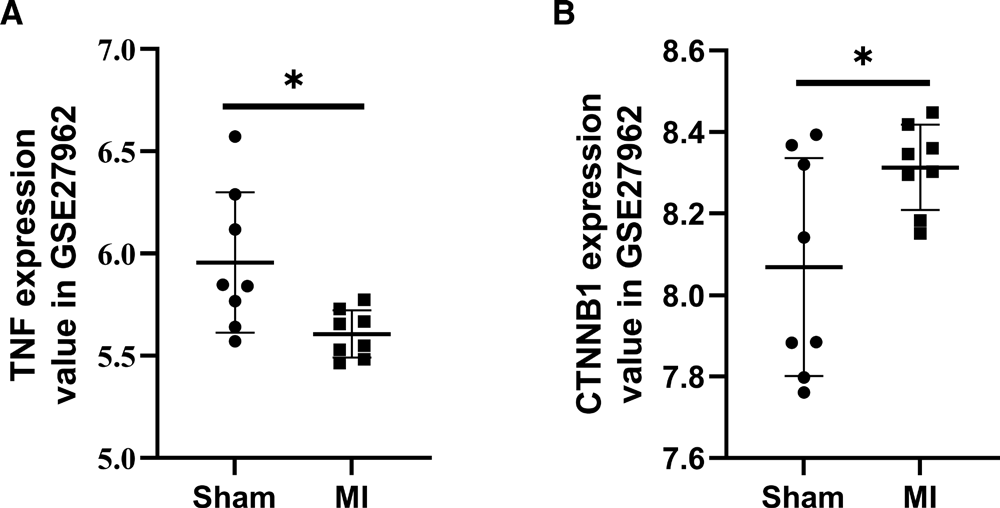
Analysis of TNF and CTNNB1 gene difference expression analysis in MI mice from GSE27962. (A) TNF expression is reduced in mice with MI. (B) Increased expression of CTNNB1 in mice with MI. MI = myocardial infarction. *P < .05.

Twelve key genes from the intersection of genes related to disorders of DSD and MI (TP53, EGFR, AKT1, IL6, TNF, STAT3, IL1B, CTNNB1, SRC, MYC, JUN, and INS) were searched and screened in GSE27962. Differential expression analysis showed that TNF was downregulated, and CTNNB1 was upregulated in the MI group (Fig. 5).

### 3.6. Molecular docking

The top 5 core target genes in the PPI network are TP53, EGFR, AKT1, IL6, and TNF, based on their degree values. The medicine DSD’s top 5 primary active ingredients had the maximum number of target genes, which were screened based on their OB and DL values in TCMSP (OB ≥ 30%, DL ≥ 0.18). We confirmed their molecular docking with the primary active ingredients. Strong binding activity is often defined as an affinity of <−5.0 kcal/mol. Additionally, when the affinity was <−7.0 kcal/mol, there was greater binding activity.

**Figure 6.**
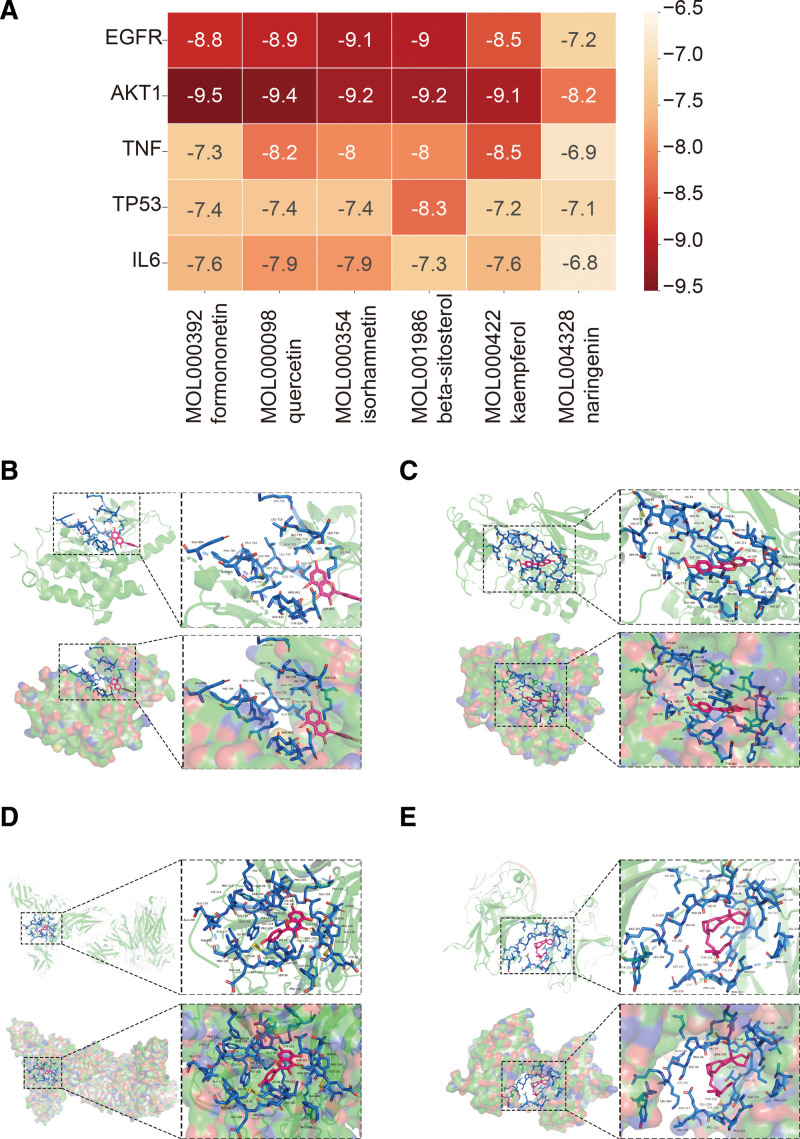
Molecular docking between main ingredients of DSD and MI-related proteins. (A) Heatmap of binding energy (kcal/mol) of core targets and active ingredients of DSD; (B) binding poses of isorhamnetin complexed with EGFR, affinity = −9.1 kcal/mol; (C) binding poses of formononetin complexed with AKT1, affinity = −9.5 kcal/mol; (D) binding poses of kaempferol complexed with TNF, affinity = −8.5 kcal/mol; (E) binding poses of beta-sitosterol complexed with TP53, affinity = −8.3 kcal/mol.

In this study, formononetin, isorhamnetin, β-sitosterol, and kaempferol exhibited strong binding activity to AKT1, EGFR, TP53, and TNF. The molecular docking results of the active ingredients TP53, EGFR, AKT1, and TNF binding strengths to the target genes are shown in Figure 6, visualized by PyMOL software. These results indicate that DSD drugs may act on AKT1, EGFR, TP53, and TNF through formononetin, isorhamnetin, β-Sitosterol, and kaempferol, thereby playing a role in treating MI.

## 4. Discussion

In the present study, we utilized network pharmacology to identify the main active ingredients and potential targets of DSD for treating MI. We then examined and confirmed the potential therapeutic benefits of the medicine for MI by utilizing molecular docking to analyze interactions between the main potential targets and active ingredients.

When MI occurs, the majority of the myocardium becomes necrotic, and the interstitium of the myocardium becomes edematous. A large number of inflammatory cells infiltrate the area, accompanied by pathological changes caused by granulation tissue. Research shows that TCM compounds play an important role in the treatment of cardiovascular disease. DSD can protect the heart and reduce myocardial cell damage after MI. Previous research has indicated that the Chinese herbs included in DSD, namely *A sinensis*, *R Cinnamomi*, *peony*, *asarum*, *G uralensis*, *tongcao*, and *jujube*, have therapeutic effects when treating MI. Research has found that the expression of lipid transport protein-2 is significantly increased in patients with cardiovascular disease and MI. After 6 weeks of intermittent training with *jujube*, researchers found that the level of the inflammatory marker lipid transport protein-2 decreased in MI rats.^[13]^ In MI rats, *A sinensis* influences the expression of vascular endothelial growth factor, promotes the proliferation of endothelial cells, and increases the number of blood vessels.^[14]^ Meanwhile, angelica polysaccharides have cardioprotective activity by reducing myocardial damage caused by ischemia-reperfusion.^[15]^ Glycyrrhizin is a flavonoid extract from *G uralensis*. Glycyrrhizin prevents myocardial fibrosis after MI by enhancing cardiac function and reducing oxidative damage and inflammatory responses. This effect may be associated with the suppression of CCL5 expression and the NF-κB pathway.^[16]^ 18β-Glycyrrrhizinic acid is from *G uralensis* and has been discovered to exhibit cardioprotective effects in AMI. These effects may be associated with the PI3K/Akt signaling pathway, which helps inhibit oxidative stress, inflammation, and apoptosis. Additionally, it aids in reducing cell contractility and Ca^2+^ concentration by acting on L-type Ca^2+^ channels.^[17]^ It has been shown that glycyrrhizic acid and *A sinensis* significantly reduced infarct size.^[18,19]^ *A sinensis* at doses of 100, 200, and 300 mg/kg decreased infarct size from 40.47 ± 4.75% to 30.71 ± 4.03%, 28.58 ± 3.11%, and 19.35 ± 1.84%, respectively (*P* < .01).^[19]^ Isoliquiritigenin is a flavonoid monomer extracted from licorice. The isoliquiritigenin treatment significantly reduces the area of MI, improves heart function, inhibits the production of reactive oxygen species, and malondialdehyde, decreases the consumption of superoxide dismutase and glutathione peroxidase, and can also notably increase nuclear Nrf2 and cytoplasmic heme oxygenase-1 levels in the infarcted myocardium, reducing oxidative stress after AMI.^[20]^ Researchers found that *G uralensis* exerts cardioprotective effects by reducing oxidative stress, enhancing endogenous antioxidants, restoring functional parameters, and maintaining structural integrity.^[21]^ According to published research, DSD has a positive adjuvant effect on coronary artery disease. It may work by regulating cholesterol metabolism, enhancing cardiac function, and increasing blood circulation. These studies have already proven the therapeutic benefits of the active pharmaceutical ingredients of DSD in the treatment of MI.

Network pharmacology can predict the pattern of drug–target interactions, provide a theoretical framework for real-world experiments, and be one of the most useful tools for predicting drug efficacy. Molecular docking can also be used to predict the interactions between active ingredient molecules and ligand proteins. After employing the network pharmacology evaluation method, 307 active ingredients from DSD were identified, along with their corresponding 5598 target genes. Among others, quercetin, kaempferol, 7-methyl-2-methyl isoflavone, spikenardin, β-sitosterol, and isorhamnetin were the active ingredients with the highest number of target interactions. According to earlier research, kaempferol and β-sitosterol have been shown to possess cardioprotective properties. Quercetin may help protect heart tissues from the damage caused by ischemia-reperfusion.^[22]^ Kaempferol is a natural antioxidant and anti-inflammatory compound found in many plants. Increasing evidence from in vitro and animal studies suggests the benefits of heart protection, demonstrating its ability to reduce oxidative stress, infarct size, and lipid distribution.^[23]^ Formononetin is a methoxyflavone abundant in many plants and herbs, which has been proven to possess various medicinal properties. Researchers have demonstrated that it can improve myocardial ischemia–reperfusion injury in rats and enhance heart function by inhibiting the activation of NLRP3 inflammasomes.^[24]^ β-Sitosterol is one of the phytosterol constituents belonging to the tetracyclic triterpenoids. Research has shown that β-sitosterol can prevent both in vitro hypoxia/reoxygenation (H/R)-induced cardiomyocyte injury and in vivo myocardial ischemia/reperfusion (I/R) injury. Its cardioprotective effects may be mediated through the regulation of myocardial I/R injury during PPARγ/NF-κB signaling.^[25]^ These studies suggest that the primary active ingredients of DSD have therapeutic effects on MI.

In the PPI network of gene targets of DSD for the treatment of MI, TP53 (p53), EGFR, AKT1, STAT3, IL6, IL1B, SRC, and MYC are in central positions, indicating that they may be key targets for MI treatment. It was found that p53-responsive microRNAs may help stratify the risk of future events in patients with ischemic heart failure as well as cardiac remodeling in postinfarction patients. The interaction of S100B and its receptor for advanced glycation end products after MI may play a role in myocyte apoptosis through the activation of ERK1/2 and p53 signaling. This receptor-mediated mechanism is particularly suitable for therapeutic intervention.^[26]^ EGFR inhibition has been identified as a cardioprotective mechanism in AMI where EGFR expression is reduced. The literature suggests that the beneficial effects of nephronectin on angiogenesis and cardiac repair after MI have been established through enhancement of the EGFR/JAK2/STAT3 signaling pathway.^[27]^ It has also been found that EGFR provides a signaling connection that multiple cardioprotective stimuli appear to converge upon, including ischemic preconditioning and a variety of G-protein-coupled receptors (opioid, muscarinic, adenosine, adrenergic, bradykinin, and sphingosine 1-phosphate), which participate in the EGFR axis (in a process known as trans-activation) in different ways involving both G-protein-dependent and G-protein-independent mechanisms to promote ischemia/infarction during and after cardiomyocyte survival.^[28]^ It has been shown in the literature that mesenchymal stem cells co-expressing Akt1 and Wnt11 exhibit higher survival and cardiac differentiation capacity under H/R conditions. They effectively ameliorate H/R-induced apoptosis of cardiomyocytes. Transplantation of mesenchymal stem cells genetically engineered with AAV-Akt1-Wnt11 is a promising therapeutic option for the treatment of AMI.^[29]^ It was found that AKT1-mediated inhibition of GSK-3 activity is essential for cardioprotection after I/R. However, in the long run, Akt1 leads to fibrosis and may exacerbate cardiac dysfunction in the postinfarction heart, demonstrating a dichotomous role of AKT1 in postinfarction cardiac remodeling.^[30]^ Z-DNA-binding protein 1 knockout mice exhibited elevated IL6 levels after MI. The upstream acute IL-6/signal transducer and activator of STAT3 pathway transmits cardiac and hepatic signals to inhibit MR (salt corticosteroid receptor) expression after MI. Some studies suggest that IL-6 is a valuable biomarker for predicting severe outcomes and identifying potential therapeutic targets.^[31]^ STAT3 regulates cardiomyocyte proliferation, differentiation, survival, oxidative stress, and metabolism.^[32]^ The cardioprotective effect of growth hormone-releasing peptides on MI-induced left ventricle injury is mediated by the activation of JAK2/STAT3 signaling and the inhibition of STAT1 signaling. Additionally, it is also partially mediated by the inhibition of cardiac IL-6.^[33]^ IL1B is a key pro-inflammatory cytokine linked to the progression of atherosclerosis and MI. It plays a role in AMI and can serve as a molecular biomarker for screening and diagnosing AMI.^[34]^ The tyrosine kinase Src (cellular-Src) in MI is related to the remodeling of connexin 43 in the border zone myocardial cells after MI.^[35]^ Inhibiting it can improve the levels and conduction velocity of connexin 43 after MI, reduce the induction of arrhythmias, and provide a new approach to alleviate arrhythmias after MI. Literature suggests that stimulating SRC with MCB-613 (and its derivatives) may be a potential therapeutic method to inhibit cardiac dysfunction after MI.^[36]^ Literature suggests that stimulating SRC with MCB-613 (and its derivatives) is a potential therapeutic approach to inhibit cardiac dysfunction after MI.^[37]^ c-Myc maintains PI3K/Akt signaling activation by forming a positive feedback loop with lncRNA small nucleolar RNA host gene 1, thereby inducing cardiomyocyte proliferation and improving cardiac function after MI. Therefore, it may be a potential therapeutic approach for post-MI cardiac dysfunction.^[38]^ It may also be a promising cardiac regeneration strategy for treating post-MI heart failure.^[38]^ Our results are consistent with previous findings, suggesting that TP53, EGFR, AKT1, STAT3, IL6, IL1B, SRC, and MYC are likely to be the core targets of DSD for the treatment of MI.

In this study, we systematically screened and investigated the different target mechanisms of DSD in the treatment of MI as well as the molecular signaling pathways of the compounds associated with their function. It has been demonstrated that both the compounds and the pharmacological effects of DSD exhibit multifaceted effects, complementary actions, and the ability to target and regulate of multiple signal pathways. The molecular docking technique provides a strategy to evaluate the binding patterns between active ingredients and disease-related targets, but potential compounds still require experimental validation. The study demonstrated that the pharmacological effects of the active ingredients of DSD in the infarction therapeutic pathway are characterized by multiple signaling pathways that target, regulate, and have various complementary effects. Due to the limitations of the network pharmacology analysis method, further studies should be conducted to identify the main pharmacologically active ingredients in the drug. Subsequent analysis and more in-depth experiments should be performed on the relevant drug targets and signaling pathways.

## 5. Conclusion

In conclusion, the treatment of MI by DSD is a multi-component, multi-pathway, multi-target, and multi-channel synergistic process. The results suggest that the active ingredients in this compound, such as formononetin, isorhamnetin, β-sitosterol, and kaempferol, may act on the target proteins TP53, TNF, EGFR, AKT1, and IL6, exerting their effects on improving MI through anti-apoptosis mechanisms and inhibition of the inflammatory response. The main mechanism of DSD treatment for MI is still unclear. This study utilized network pharmacology and molecular docking to predict the most likely targets and active ingredients of DSD for the treatment of MI, providing fundamental research data to explore the mechanism of DSD for the treatment of MI.

## Author contributions

**Conceptualization:** Zhenzhen Li, Bing Li.

**Data curation:** Zhenzhen Li.

**Funding acquisition:** Bing Li.

**Methodology:** Zhenzhen Li, Shuang Liu, Rui Zhang.

**Resources:** Zhenzhen Li, Shuang Liu, Rui Zhang.

**Software:** Zhenzhen Li, Shuang Liu, Rui Zhang.

**Supervision:** Bing Li.

**Writing – original draft:** Zhenzhen Li, Shuang Liu, Rui Zhang.

**Writing – review & editing:** Bing Li.

## Supplementary Material


