# Supplementary material for: Exploring the mechanism of Danggui Sini Decoction in the treatment of myocardial infarction: A systematic review, network pharmacology, and molecular docking
Source: Medicine (Baltimore). 2024 Oct 18;103(42):e40073. doi: 10.1097/MD.0000000000040073 (PMC11495767; doi:10.1097/MD.0000000000040073)

**Supplemental Table 1 The active ingredients of DSD were screened**

| No. | Compound | Herb | Number of targets | Databases |
| --- | --- | --- | --- | --- |
| 1 | quercetin | Gancao, Dazao | 154 | TCMSP |
| 2 | Cetylic Acid,Hexadecanoic Acid,Palmitic Acid | Danggui, Dazao | 86 | TCMIP |
| 3 | Myristic Acid | Danggui, Dazao | 86 | TCMIP |
| 4 | Lignoceric Acid | Danggui | 86 | TCMIP |
| 5 | Octadecanoic?Acid,Stearic Acid | Dazao | 86 | TCMIP |
| 6 | L-Î‘-Amino-Î”-Hydroxyvaleric Acid | Shaoyao | 85 | TCMIP |
| 7 | Oleanolic Acid | Dazao | 75 | TCMIP |
| 8 | Betulinic Acid | Dazao | 75 | TCMIP |
| 9 | Cis-9,Cis-12-Linoleic Acid,Inositol,Linoleic,Linoleic Acid | Dazao | 64 | TCMIP |
| 10 | Palmitoleic Acid,9-Hexadecenoic Acid | Dazao | 64 | TCMIP |
| 11 | kaempferol | Shaoyao | 63 | TCMSP |
| 12 | 3,3'-Dimethylquercetin | Gancao | 51 | TCMIP |
| 13 | 3',7-Dihydroxy-4',6-Dimethoxyisoflavone | Gancao | 51 | TCMIP |
| 14 | Kumatakenin | Gancao | 51 | TCMIP |
| 15 | Casuariin | Shaoyao | 50 | TCMIP |
| 16 | 5-Desgalloylstachyurin | Shaoyao | 50 | TCMIP |
| 17 | 13-Methyl Tetradecanoic Acid | Shaoyao | 49 | TCMIP |
| 18 | Glycyrrhetinic Acid | Gancao | 48 | TCMIP |
| 19 | Desmosterol | Dazao | 47 | TCMIP |
| 20 | Sitosterol,Î’-Sitosterol | Guizhi, Shaoyao, Gancao, Dazao | 44 | TCMIP |
| 21 | (S)-5,7-Dihydroxy-2-Phenylchroman-4-One,Pinocembrin | Gancao | 44 | TCMIP |
| 22 | 7-Methoxy-2-methyl isoflavone | Gancao | 43 | TCMSP |
| 23 | Liquiritigenin | Gancao | 42 | TCMIP |
| 24 | Catechin | Shaoyao, Dazao | 40 | TCMIP |
| 25 | Zizybeosidei | Dazao | 40 | TCMIP |
| 26 | Zizybeosideii | Dazao | 40 | TCMIP |
| 27 | formononetin | Gancao | 39 | TCMSP, TCMIP |
| 28 | beta-sitosterol | Danggui, Guizhi, Shaoyao, Dazao | 38 | TCMSP |
| 29 | Nuciferine | Dazao | 38 | TCMIP |
| 30 | isorhamnetin | Gancao | 37 | TCMSP |
| 31 | naringenin | Gancao | 37 | TCMSP |
| 32 | Sucrose | Dazao | 37 | TCMIP |
| 33 | D-Glucose,Glucose | Dazao | 36 | TCMIP |
| 34 | 1-O-Galloyl-Glucose | Shaoyao | 35 | TCMIP |
| 35 | Medicarpin | Gancao | 34 | TCMSP |
| 36 | Alphitolic Acid | Dazao | 33 | TCMIP |
| 37 | Succinic Acid | Danggui | 33 | TCMIP |
| 38 | Sebacic Acid | Danggui | 33 | TCMIP |
| 39 | Azelaic Acid | Danggui | 33 | TCMIP |
| 40 | licochalcone a | Gancao | 32 | TCMSP |
| 41 | Malic Acid | Dazao | 32 | TCMIP |
| 42 | Isoliquiritin | Gancao | 32 | TCMIP |
| 43 | Neoisoliquiritin | Gancao | 32 | TCMIP |
| 44 | Stigmasterol | Danggui, Dazao | 31 | TCMSP, TCMIP |
| 45 | 2-[(3R)-8,8-dimethyl-3,4-dihydro-2H-pyrano[6,5-f]chromen-3-yl]-5-methoxyphenol | Gancao | 31 | TCMSP |
| 46 | Proanthocyanidin B2,Procyanidinb2 | Guizhi | 31 | TCMIP |
| 47 | Palbinone | Shaoyao | 31 | TCMIP |
| 48 | shinpterocarpin | Gancao | 30 | TCMSP |
| 49 | Vestitol | Gancao | 30 | TCMSP |
| 50 | 1-Methoxyphaseollidin | Gancao | 29 | TCMSP |
| 51 | Licoagrocarpin | Gancao | 29 | TCMSP |
| 52 | Stepholidine | Dazao | 29 | TCMSP |
| 53 | Isoliquiritigenin | Gancao | 29 | TCMIP |
| 54 | 2,4,4'-Trihydroxychalcone | Gancao | 29 | TCMIP |
| 55 | Cryptopin | Xixin | 28 | TCMSP |
| 56 | 3'-Hydroxy-4'-O-Methylglabridin | Gancao | 28 | TCMSP |
| 57 | 3'-Methoxyglabridin | Gancao | 28 | TCMSP, TCMIP |
| 58 | Fumarine | Dazao | 28 | TCMSP |
| 59 | Glypallichalcone | Gancao | 27 | TCMSP |
| 60 | HMO | Gancao | 27 | TCMSP |
| 61 | stepharine | Dazao | 27 | TCMSP, TCMIP |
| 62 | Nuciferin | Dazao | 27 | TCMSP |
| 63 | Casuarictin | Shaoyao | 27 | TCMIP |
| 64 | Eugeniin | Shaoyao | 27 | TCMIP |
| 65 | Fructose | Dazao | 27 | TCMIP |
| 66 | (3S)-7-hydroxy-3-(2,3,4-trimethoxyphenyl)chroman-4-one | Xixin | 26 | TCMSP |
| 67 | Glepidotin A | Gancao | 26 | TCMSP |
| 68 | Glyasperins M | Gancao | 26 | TCMSP |
| 69 | Riboflavine | Dazao | 26 | TCMIP |
| 70 | Glabridin | Gancao | 25 | TCMSP |
| 71 | 7-Acetoxy-2-methylisoflavone | Gancao | 25 | TCMSP |
| 72 | Anhydrocinnzeylanine | Guizhi | 25 | TCMIP |
| 73 | Strictinin | Shaoyao | 25 | TCMIP |
| 74 | Tellimagrandin I | Shaoyao | 25 | TCMIP |
| 75 | Glyasperin C | Gancao | 24 | TCMSP |
| 76 | 3-O-Acetyl-Glycyrrhetinic Acid | Gancao | 24 | TCMIP |
| 77 | sesamin | Xixin | 23 | TCMSP, TCMIP |
| 78 | 3-(2,4-dihydroxyphenyl)-8-(1,1-dimethylprop-2-enyl)-7-hydroxy-5-methoxy-coumarin | Gancao | 23 | TCMSP |
| 79 | (S)-Coclaurine | Dazao | 23 | TCMSP |
| 80 | Pedunculagin | Shaoyao | 23 | TCMIP |
| 81 | Calycosin | Gancao | 22 | TCMSP |
| 82 | Phaseolinisoflavan | Gancao | 22 | TCMSP |
| 83 | Gancaonin B | Gancao | 22 | TCMSP, TCMIP |
| 84 | 4-Hydroxycoumarin,Folinic Acid | Danggui | 22 | TCMIP |
| 85 | 3-O-[Î’-D-Glucuronopyranosyl-(1â†’2)-O-Î’-D-Glucuronopyranosyl]-24-Hydroxyglabrolide | Gancao | 22 | TCMIP |
| 86 | Lupiwighteone | Gancao | 21 | TCMSP, TCMIP |
| 87 | glyasperin B | Gancao | 21 | TCMSP |
| 88 | kanzonols W | Gancao | 21 | TCMSP |
| 89 | (2S)-6-(2,4-dihydroxyphenyl)-2-(2-hydroxypropan-2-yl)-4-methoxy-2,3-dihydrofuro[3,2-g]chromen-7-one | Gancao | 21 | TCMSP |
| 90 | Glabrone | Gancao | 21 | TCMSP |
| 91 | Dibutyl Uralsaponin A Ester | Gancao | 21 | TCMIP |
| 92 | Ethyl-N-Buthy-Uralsaponin A Esters | Gancao | 21 | TCMIP |
| 93 | (E)-1-(2,4-dihydroxyphenyl)-3-(2,2-dimethylchromen-6-yl)prop-2-en-1-one | Gancao | 20 | TCMSP |
| 94 | Gancaonin A | Gancao | 20 | TCMSP, TCMIP |
| 95 | 5,7-dihydroxy-3-(4-methoxyphenyl)-8-(3-methylbut-2-enyl)chromone | Gancao | 20 | TCMSP |
| 96 | licoisoflavanone | Gancao | 20 | TCMSP |
| 97 | Gancaonin G | Gancao | 20 | TCMSP |
| 98 | Odoratin | Gancao | 20 | TCMSP |
| 99 | Carvacrol | Danggui, Xixin | 20 | TCMIP |
| 100 | (R)-Zizyphusine | Dazao | 20 | TCMIP |
| 101 | Licochalcone B | Gancao | 19 | TCMSP |
| 102 | Licoisoflavone | Gancao | 19 | TCMSP, TCMIP |
| 103 | Glabrene | Gancao | 19 | TCMSP |
| 104 | Eurycarpin A | Gancao | 19 | TCMSP |
| 105 | O-Cresol | Danggui | 19 | TCMIP |
| 106 | Stigmasterol-Î’-D-Glucoside | Danggui | 19 | TCMIP |
| 107 | Alexandrin,Daucosterol,Eleutheroside A | Shaoyao | 19 | TCMIP |
| 108 | Alexandrin,Daucosterol,Caproic Acid,Eleutheroside A,Sitogluside,Strumaroside,Î’-Sitosterol-Î’-D-Glucoside | Shaoyao | 19 | TCMIP |
| 109 | Methyl-N-Butyl-Uralsaponin A Esters | Gancao | 19 | TCMIP |
| 110 | 3-Oxo-Olean-12-En-28-Oic Acid | Dazao | 19 | TCMIP |
| 111 | glyasperin F | Gancao | 18 | TCMSP |
| 112 | 3-(3,4-dihydroxyphenyl)-5,7-dihydroxy-8-(3-methylbut-2-enyl)chromone | Gancao | 18 | TCMSP |
| 113 | Glyzaglabrin | Gancao | 18 | TCMSP, TCMIP |
| 114 | Licoagroisoflavone | Gancao | 18 | TCMSP, TCMIP |
| 115 | dehydroglyasperins C | Gancao | 18 | TCMSP |
| 116 | Betulonic Acid | Dazao | 18 | TCMIP |
| 117 | Jujuboside A | Dazao | 18 | TCMIP |
| 118 | Retinol | Danggui | 18 | TCMIP |
| 119 | 2,3-Dicresol | Danggui | 18 | TCMIP |
| 120 | Anhydrocinnzeylanol | Guizhi | 18 | TCMIP |
| 121 | 2-Isopropyl-5-Methylanisole | Xixin | 18 | TCMIP |
| 122 | Licoricesaponin A3 | Gancao | 18 | TCMIP |
| 123 | Licoricesaponin G2 | Gancao | 18 | TCMIP |
| 124 | Licoricesaponin J2 | Gancao | 18 | TCMIP |
| 125 | Licoricesaponin K2 | Gancao | 18 | TCMIP |
| 126 | Jujuboside B | Dazao | 18 | TCMIP |
| 127 | Jujuboside B1 | Dazao | 18 | TCMIP |
| 128 | Zizyphussaponin I | Dazao | 18 | TCMIP |
| 129 | Zizyphussaponin Ii | Dazao | 18 | TCMIP |
| 130 | Zizyphussaponin Iii | Dazao | 18 | TCMIP |
| 131 | Inermine | Gancao | 17 | TCMSP |
| 132 | Semilicoisoflavone B | Gancao | 17 | TCMSP, TCMIP |
| 133 | licochalcone G | Gancao | 17 | TCMSP |
| 134 | Glycyrin | Gancao | 17 | TCMSP, TCMIP |
| 135 | Licoisoflavone B | Gancao | 17 | TCMSP, TCMIP |
| 136 | Quercetin der. | Gancao | 17 | TCMSP |
| 137 | Glycyrrhiza flavonol A | Gancao | 17 | TCMSP |
| 138 | berberine | Dazao | 17 | TCMSP, TCMIP |
| 139 | Glycyrrhizic Acid | Gancao | 17 | TCMIP |
| 140 | Licoricesaponin B2 | Gancao | 17 | TCMIP |
| 141 | Licoricesaponin C2 | Gancao | 17 | TCMIP |
| 142 | Licoricesaponin H2 | Gancao | 17 | TCMIP |
| 143 | Methyl 18Î‘-Hydroxyglycyrrhetate | Gancao | 17 | TCMIP |
| 144 | Uralsaponin A | Gancao | 17 | TCMIP |
| 145 | Uralsaponin B | Gancao | 17 | TCMIP |
| 146 | Jujubasaponin Iv | Dazao | 17 | TCMIP |
| 147 | Jujubasaponin V | Dazao | 17 | TCMIP |
| 148 | Ziziphin | Dazao | 17 | TCMIP |
| 149 | licopyranocoumarin | Gancao | 16 | TCMSP, TCMIP |
| 150 | Methylglycyrrhetate | Gancao | 16 | TCMIP |
| 151 | Methyl-24-Hydroxyglycyrrhetate | Gancao | 16 | TCMIP |
| 152 | Glepidotin B | Gancao | 15 | TCMSP |
| 153 | Licoricone | Gancao | 15 | TCMSP, TCMIP |
| 154 | 2-(3,4-dihydroxyphenyl)-5,7-dihydroxy-6-(3-methylbut-2-enyl)chromone | Gancao | 15 | TCMSP |
| 155 | (2R)-7-hydroxy-2-(4-hydroxyphenyl)chroman-4-one | Gancao | 15 | TCMSP |
| 156 | Isolicoflavonol | Gancao | 15 | TCMSP, TCMIP |
| 157 | Inflacoumarin A | Gancao | 15 | TCMSP |
| 158 | 7,2',4'-trihydroxy－5-methoxy-3－arylcoumarin | Gancao | 15 | TCMSP |
| 159 | Pavilion,Scopoletin,Scopoletol,Trigonelline | Danggui | 15 | TCMIP |
| 160 | 3-O-(Cis-P-Coumaroyl)-Maslinic Acid | Dazao | 15 | TCMIP |
| 161 | 3-O-(Trans-P-Coumaroyl)-Maslinic Acid | Dazao | 15 | TCMIP |
| 162 | Isotrifoliol | Gancao | 14 | TCMSP, TCMIP |
| 163 | Phaseol | Gancao | 14 | TCMSP, TCMIP |
| 164 | Formononetin-7-Glucoside | Gancao | 14 | TCMIP |
| 165 | Isoononin | Gancao | 14 | TCMIP |
| 166 | Narcissin | Gancao | 14 | TCMIP |
| 167 | Ordoritin-Glucoside | Gancao | 14 | TCMIP |
| 168 | 2Î‘,3Î’-Dihydroxyolean-12-En-28-Oic Acid | Dazao | 14 | TCMIP |
| 169 | Jaranol | Gancao | 13 | TCMSP |
| 170 | Liquiritigenin-7,4'-Diglucoside | Gancao | 13 | TCMIP |
| 171 | taxifolin | Guizhi | 12 | TCMSP |
| 172 | DFV | Gancao | 12 | TCMSP |
| 173 | (2S)-2-[4-hydroxy-3-(3-methylbut-2-enyl)phenyl]-8,8-dimethyl-2,3-dihydropyrano[2,3-f]chromen-4-one | Gancao | 12 | TCMSP |
| 174 | (E)-3-[3,4-dihydroxy-5-(3-methylbut-2-enyl)phenyl]-1-(2,4-dihydroxyphenyl)prop-2-en-1-one | Gancao | 12 | TCMSP |
| 175 | (2S)-7-hydroxy-2-(4-hydroxyphenyl)-8-(3-methylbut-2-enyl)chroman-4-one | Gancao | 12 | TCMSP |
| 176 | Gancaonin H | Gancao | 12 | TCMSP |
| 177 | (+)-catechin | Guizhi, Shaoyao, Dazao | 11 | TCMSP |
| 178 | Glycyrol | Gancao | 11 | TCMSP, TCMIP |
| 179 | Glabranin | Gancao | 11 | TCMSP |
| 180 | (-)-catechin | Dazao | 11 | TCMSP |
| 181 | Glabrolide | Gancao | 11 | TCMIP |
| 182 | Methyl-24-Hydroxy-11-Deoxoglycyrrhetate | Gancao | 11 | TCMIP |
| 183 | Colubrinic Acid | Dazao | 11 | TCMIP |
| 184 | euchrenone | Gancao | 10 | TCMSP |
| 185 | 1,3-dihydroxy-9-methoxy-6-benzofurano[3,2-c]chromenone | Gancao | 10 | TCMSP |
| 186 | Vitamin B12 | Danggui | 10 | TCMIP |
| 187 | Licoricesaponin D3 | Gancao | 10 | TCMIP |
| 188 | Licoricesaponin E2 | Gancao | 10 | TCMIP |
| 189 | Licoricesaponin F3 | Gancao | 10 | TCMIP |
| 190 | 3-O-(Cis-P-Coumaroyl)-Alphitolic Acid | Dazao | 10 | TCMIP |
| 191 | 3-O-(Trans-P-Coumaroyl)-Alphitolic Acid | Dazao | 10 | TCMIP |
| 192 | Jujubasaponin Vi | Dazao | 10 | TCMIP |
| 193 | Swertisin | Dazao | 10 | TCMIP |
| 194 | 4,9-dimethoxy-1-vinyl-$b-carboline | Xixin | 9 | TCMSP |
| 195 | 1,3-dihydroxy-8,9-dimethoxy-6-benzofurano[3,2-c]chromenone | Gancao | 9 | TCMSP |
| 196 | 3,4,5-Trihydroxybenzoic Acid,Gallic Acid,Gallicacidmonohydrate | Shaoyao | 9 | TCMIP |
| 197 | Aminobutyl Canavalmine | Shaoyao | 9 | TCMIP |
| 198 | Isoquercitrin,Isoquercetrin,Kuwanon H | Gancao | 9 | TCMIP |
| 199 | Neoliquiritin | Gancao | 9 | TCMIP |
| 200 | Uralenneoside | Gancao | 9 | TCMIP |
| 201 | Choerospondin | Gancao | 9 | TCMIP |
| 202 | Asparagine | Dazao | 9 | TCMIP |
| 203 | Betulin | Dazao | 9 | TCMIP |
| 204 | Caribine | Xixin | 8 | TCMSP |
| 205 | Kanzonol F | Gancao | 8 | TCMSP |
| 206 | 6-prenylated eriodictyol | Gancao | 8 | TCMSP, TCMIP |
| 207 | 8-prenylated eriodictyol | Gancao | 8 | TCMSP, TCMIP |
| 208 | Xambioona | Gancao | 8 | TCMSP, TCMIP |
| 209 | coumestrol | Dazao | 8 | TCMSP |
| 210 | Rutin,Rutoside,Vitamin P | Gancao, Dazao | 8 | TCMIP |
| 211 | Uracil | Danggui | 8 | TCMIP |
| 212 | Coumarin | Guizhi | 8 | TCMIP |
| 213 | Z-1S,5R-Î’-Pinen-10-Yl-Î’-Vicianoside | Shaoyao | 8 | TCMIP |
| 214 | Paeonianin A | Shaoyao | 8 | TCMIP |
| 215 | Paeonianin B | Shaoyao | 8 | TCMIP |
| 216 | Paeonianin C | Shaoyao | 8 | TCMIP |
| 217 | Paeonianin D | Shaoyao | 8 | TCMIP |
| 218 | 1,2,6-Tri-O-Galloyl-Î’-D-Glucose | Shaoyao | 8 | TCMIP |
| 219 | 1,3,6-Trigalloyl-Î’-D-Glucose | Shaoyao | 8 | TCMIP |
| 220 | Acetylborneol,Bornyl Acetate,L-Bornyl Acetate | Xixin | 8 | TCMIP |
| 221 | Nicotiflorin | Gancao | 8 | TCMIP |
| 222 | Licoagroside C | Gancao | 8 | TCMIP |
| 223 | Tartaric Acid | Dazao | 8 | TCMIP |
| 224 | Licocoumarone | Gancao | 7 | TCMSP, TCMIP |
| 225 | Isoglycyrol | Gancao | 7 | TCMSP, TCMIP |
| 226 | Vanillin | Danggui | 7 | TCMIP |
| 227 | Choline | Danggui | 7 | TCMIP |
| 228 | Benzoic Acid | Shaoyao | 7 | TCMIP |
| 229 | 2,3-O-(S)-Hexahydroxydiphenoyl-D-Glucopyranose | Shaoyao | 7 | TCMIP |
| 230 | Peonin | Shaoyao | 7 | TCMIP |
| 231 | Glyuranolide | Gancao | 7 | TCMIP |
| 232 | Spinosin | Dazao | 7 | TCMIP |
| 233 | ent-Epicatechin | Guizhi | 6 | TCMSP |
| 234 | 8-(6-hydroxy-2-benzofuranyl)-2,2-dimethyl-5-chromenol | Gancao | 6 | TCMSP |
| 235 | liquiritin | Gancao | 6 | TCMSP, TCMIP |
| 236 | Sigmoidin-B | Gancao | 6 | TCMSP |
| 237 | Anisic Acid | Danggui | 6 | TCMIP |
| 238 | Cinnamic Acid | Guizhi | 6 | TCMIP |
| 239 | 1,2,3-Tri-O-Galloyl-Î’-D-Glucose | Shaoyao | 6 | TCMIP |
| 240 | Canavalia Gibberellin Ii | Shaoyao | 6 | TCMIP |
| 241 | 6,8-Bis(C-Î’-Glucosyl)-Apigenin,Vicenin-2 | Gancao | 6 | TCMIP |
| 242 | Isoschaftoside | Gancao | 6 | TCMIP |
| 243 | Violanthin | Gancao | 6 | TCMIP |
| 244 | Isoviolanthin | Gancao | 6 | TCMIP |
| 245 | 6'-P-Coumaroylspinosin | Dazao | 6 | TCMIP |
| 246 | 6'-Feruloylspinosin | Dazao | 6 | TCMIP |
| 247 | 6'-Sinapoylspinosin | Dazao | 6 | TCMIP |
| 248 | 5-Hydroxycoumarin,Guaiacol | Danggui | 5 | TCMIP |
| 249 | Brefeldin A | Danggui | 5 | TCMIP |
| 250 | Isoeugenol | Danggui | 5 | TCMIP |
| 251 | Paeonianiin E | Shaoyao | 5 | TCMIP |
| 252 | Paeonol,Scopoletin | Shaoyao | 5 | TCMIP |
| 253 | 1,2,4,6-Tetra-O-Galloyl-Î’-D-Glucose | Shaoyao | 5 | TCMIP |
| 254 | Eugenol,Guaiacol | Xixin | 5 | TCMIP |
| 255 | Liquiritigenin 4'-O-Î’-D-Apio-D-Furanosyl(1â†’2)-Î’-D-Glucopyranoside | Gancao | 5 | TCMIP |
| 256 | (-)-taxifolin | Guizhi | 4 | TCMSP |
| 257 | paeoniflorin | Shaoyao | 4 | TCMSP, TCMIP |
| 258 | ZINC05223929 | Xixin | 4 | TCMSP |
| 259 | Phenol | Danggui, Shaoyao | 4 | TCMIP |
| 260 | 2',4'-Dihydroxyacetophenone | Danggui | 4 | TCMIP |
| 261 | Eugenol Methyl Ether,Methyl Isoeugenol,Methyleugenol | Xixin | 4 | TCMIP |
| 262 | Niacin,Nicotinic Acid | Dazao | 4 | TCMIP |
| 263 | sitosterol | Guizhi, Shaoyao, Gancao, Tongcao | 3 | TCMSP |
| 264 | [(1S)-3-[(E)-but-2-enyl]-2-methyl-4-oxo-1-cyclopent-2-enyl] (1R,3R)-3-[(E)-3-methoxy-2-methyl-3-oxoprop-1-enyl]-2,2-dimethylcyclopropane-1-carboxylate | Xixin | 3 | TCMSP |
| 265 | Mauritine D | Dazao | 3 | TCMSP |
| 266 | Albiflorin | Shaoyao | 3 | TCMIP |
| 267 | Oxypaeoniflorin | Shaoyao | 3 | TCMIP |
| 268 | 7-Hydroxycoumarin,Skimmetin,Umbelliferone | Danggui | 3 | TCMIP |
| 269 | P-Cresol | Danggui | 3 | TCMIP |
| 270 | Homosenkyunolide H | Danggui | 3 | TCMIP |
| 271 | Homosenkyunolide I | Danggui | 3 | TCMIP |
| 272 | Dihydromelilotoside | Guizhi | 3 | TCMIP |
| 273 | Paeonioflorin | Shaoyao | 3 | TCMIP |
| 274 | (3S,5R,8R,9R,10S,14S)-3,17-dihydroxy-4,4,8,10,14-pentamethyl-2,3,5,6,7,9-hexahydro-1H-cyclopenta[a]phenanthrene-15,16-dione | Shaoyao | 2 | TCMSP |
| 275 | (-)-Medicocarpin | Gancao | 2 | TCMSP |
| 276 | paryriogenin I | Tongcao | 2 | TCMSP |
| 277 | Ruvoside_qt | Dazao | 2 | TCMSP |
| 278 | beta-carotene | Dazao | 2 | TCMSP |
| 279 | 3-O-trans ferulylquinic acid | Danggui | 2 | TCMIP |
| 280 | 1-Tetradecanol | Danggui | 2 | TCMIP |
| 281 | Dodecanol | Danggui | 2 | TCMIP |
| 282 | 3,4-Dihydroxybenzoic Acid,Protocatechuic Acid | Guizhi | 2 | TCMIP |
| 283 | 1,2,3,4,6-Pentagalloylglucose | Shaoyao | 2 | TCMIP |
| 284 | Lysicamine | Dazao | 2 | TCMIP |
| 285 | Vitamin B1 | Dazao | 2 | TCMIP |
| 286 | paeoniflorgenone | Shaoyao | 1 | TCMSP |
| 287 | Mairin | Shaoyao, Xixin, Gancao, Dazao | 1 | TCMSP |
| 288 | icos-5-enoic acid | Gancao | 1 | TCMSP |
| 289 | gadelaidic acid | Gancao | 1 | TCMSP |
| 290 | Tetrapanoside B_qt | Tongcao | 1 | TCMSP |
| 291 | zizyphus saponin I_qt | Dazao | 1 | TCMSP |
| 292 | Daechuine S6 | Dazao | 1 | TCMSP |
| 293 | Jujubasaponin V_qt | Dazao | 1 | TCMSP |
| 294 | Maruzen M,P-Ethylphenol | Danggui | 1 | TCMIP |
| 295 | 2-Methyl-Dodecane-5-One | Danggui | 1 | TCMIP |
| 296 | 1,1,5-Trimethyl-2-Formyl-Cyclohexa-2,5-Diene-4-One | Danggui | 1 | TCMIP |
| 297 | Canavalia Gibberellin I | Shaoyao | 1 | TCMIP |
| 298 | (R)-P-Menth-1-En-4-Ol,Terpinen-4-Ol | Xixin | 1 | TCMIP |
| 299 | Isoasarone,Î‘-Asarone | Xixin | 1 | TCMIP |
| 300 | Corylifolinin,Isobavachalcone | Gancao | 1 | TCMIP |
| 301 | (E)-1-[2,4-Dihydroxy-3-(3-Methyl-2-Butenyl)Phenyl]-3-(4-Hydroxy-3-[3-Methyl-2-Butenyl)Phenyl]-2-Propen-1-One | Gancao | 1 | TCMIP |
| 302 | Gancaonin F | Gancao | 1 | TCMIP |
| 303 | Licofuranocoumarin | Gancao | 1 | TCMIP |
| 304 | Erythrinin C | Gancao | 1 | TCMIP |
| 305 | Licoagrochalcone A | Gancao | 1 | TCMIP |
| 306 | Asimilobine | Dazao | 1 | TCMIP |
| 307 | Vomifoliol | Dazao | 1 | TCMIP |

**Supplemental Table 2 Compounds of DSD from HERB database.**

| Ingredient id | Ingredient name | herb |
| --- | --- | --- |
| HBIN000322 | 1,1,5-trimethyl-2-formyl-cyclohexa-2,5-diene-4-one | Danggui |
| HBIN000721 | 1,2-benzenedicarboxylic acid | Danggui |
| HBIN000821 | 1,2-dimethylbenzene | Danggui |
| HBIN001584 | 1,5,5,6-tetramethyl-1,3-Cyclohexadiene | Danggui |
| HBIN002497 | 1-dodecene | Danggui |
| HBIN002579 | 1-hexadecanol | Danggui |
| HBIN002740 | 1-methyl-2-dodecyl-4-(1h)-quinolone | Danggui |
| HBIN003078 | (1R,2S,4R)-1-ethyl-1-methyl-2,4-bis(1-methylethyl)cyclohexane | Danggui |
| HBIN003129 | (1R,4R,5S)-4-isopropenyl-1,8-dimethylspiro[4.5]dec-8-ene | Danggui |
| HBIN003261 | (1S,4aR,8aR)-1-isopropyl-7-methyl-4-methylene-2,3,4a,5,6,8a-hexahydro-1H-naphthalene | Danggui |
| HBIN003357 | 1-tridecene | Danggui |
| HBIN003962 | 2,3-dicresol | Danggui |
| HBIN004186 | 24,24-dimethyl-5alpha-cholesta-8-en-3beta-ol | Danggui |
| HBIN004236 | 2,4,5-trimethylbenzaldehyde | Danggui |
| HBIN004254 | 2,4,6-trimethylbenzaldehyde | Danggui |
| HBIN004256 | 2,4,6-trimethyl-Octane | Danggui |
| HBIN004333 | 2',4'-dihydroxyacetophenone | Danggui |
| HBIN004354 | 2,4-dimethylbenzaldehyde | Danggui |
| HBIN004555 | 2,4-Xylylaldehyde | Danggui |
| HBIN004655 | 2,5-dimethylbenzaldehyde | Danggui |
| HBIN004942 | 2,6-di(phenyl)thiopyran-4-thione | Danggui |
| HBIN005965 | 2-methyl-3-buten-2-ol | Danggui |
| HBIN005997 | 2-methyl-5-decanone | Danggui |
| HBIN006045 | 2-methyldodecan-5-one | Danggui |
| HBIN006046 | 2-methyl-dodecane-5-one | Danggui |
| HBIN006065 | 2-Methylhexadecanoic acid | Danggui |
| HBIN006301 | 2-pentanoylbenzoic acid | Danggui |
| HBIN006852 | 2-valerylbenzoic acid | Danggui |
| HBIN007383 | 3,4-dihydroxyallylbenzene 4-o-[alpha-l-rhamnopyranosyl-(1-6)]-beta-d-glucopyranoside | Danggui |
| HBIN007446 | 3,4-DIMETHYLBENZALDEHYDE | Danggui |
| HBIN007807 | 3,7-dimethylnonane | Danggui |
| HBIN008360 | 3-Butylidene-7-hydroxyphthalide | Danggui |
| HBIN008361 | 3-butylidene-phalide | Danggui |
| HBIN008448 | (3E)-3-butylidene-7-hydroxy-2-benzofuran-1-one | Danggui |
| HBIN008918 | 3-methylbutyl-benzene | Danggui |
| HBIN009611 | (3S)-3-butyl-3H-isobenzofuran-1-one | Danggui |
| HBIN009612 | 3(S)-3-Butyl-4,5-dihydrophthalide | Danggui |
| HBIN010297 | 4-chloro-N-[1-methyl-5-[[1-methyl-5-[[1-methyl-5-(2-morpholinoethylcarbamoyl)pyrrol-3-yl]carbamoyl]pyrrol-3-yl]carbamoyl]pyrrol-3-yl]-5-[2-(2-pyridyl)ethylamino]isothiazole-3-carboxamide | Danggui |
| HBIN010394 | 4-ethylresorcinol | Danggui |
| HBIN010673 | 4-Methyl-6-hepten-3-one | Danggui |
| HBIN010762 | 4-Octanone | Danggui |
| HBIN011719 | 5-Indolol | Danggui |
| HBIN011869 | 5-o-methylvisamminol | Danggui |
| HBIN012046 | 6,7,3',8'-diligustilide | Danggui |
| HBIN012338 | 6-Ethylresorcinol | Danggui |
| HBIN012667 | 6-O-E-Feruloylajugol | Danggui |
| HBIN012771 | (6R)-6-butylcyclohepta-1,4-diene | Danggui |
| HBIN012833 | 6-undecanol | Danggui |
| HBIN012834 | 6-undecanone | Danggui |
| HBIN012857 | 7,10-PENTADECADIYNOIC ACID | Danggui |
| HBIN013503 | 80-57-9 | Danggui |
| HBIN014593 | Acoradiene | Danggui |
| HBIN014684 | adenine | Danggui |
| HBIN014685 | adeninenucleoside | Danggui |
| HBIN015219 | Allocymene | Danggui |
| HBIN015235 | alloocimene | Danggui |
| HBIN015333 | α-acoradiene | Danggui |
| HBIN015448 | α-cedrene | Danggui |
| HBIN015451 | alpha-chamigrene | Danggui |
| HBIN015462 | α-copaene | Danggui |
| HBIN015949 | Amyl ketone | Danggui |
| HBIN016086 | angelicide | Danggui |
| HBIN016122 | anglica polysacharide | Danggui |
| HBIN016183 | anisicacid | Danggui |
| HBIN016201 | ANN | Danggui |
| HBIN016886 | aromadendrene | Danggui |
| HBIN017449 | azelaic acid | Danggui |
| HBIN017450 | Azelex | Danggui |
| HBIN017664 | BdPh | Danggui |
| HBIN017903 | bergamotene | Danggui |
| HBIN017953 | β-acoradiene | Danggui |
| HBIN018012 | beta-caryophyllene | Danggui |
| HBIN018197 | beta-myrcene | Danggui |
| HBIN018467 | bicycloelemene | Danggui |
| HBIN018821 | brefeldin a | Danggui |
| HBIN018952 | BUA | Danggui |
| HBIN019062 | Butal | Danggui |
| HBIN019064 | butanal | Danggui |
| HBIN019073 | butanoic acid | Danggui |
| HBIN019492 | campheoside i | Danggui |
| HBIN019506 | camphoricacid | Danggui |
| HBIN019507 | ()-Camphoric acid | Danggui |
| HBIN020406 | choline | Danggui |
| HBIN020427 | Chrysanthemaxanthin | Danggui |
| HBIN020868 | cis-Isoeugenol | Danggui |
| HBIN020876 | cis-ligustilide | Danggui |
| HBIN021167 | Cnidilide | Danggui |
| HBIN021168 | Cnidilin | Danggui |
| HBIN021368 | Coniferyl ferulate | Danggui |
| HBIN021686 | crinamine | Danggui |
| HBIN021898 | cumaldehyde | Danggui |
| HBIN022899 | Decanal | Danggui |
| HBIN022905 | decanoic acid | Danggui |
| HBIN023189 | delta-acoradiene | Danggui |
| HBIN023522 | D-Galacturonic acid, homopolymer | Danggui |
| HBIN023701 | dictamnine | Danggui |
| HBIN023962 | dihydropinosylvin | Danggui |
| HBIN024056 | dimethyl azelate | Danggui |
| HBIN024057 | dimethyl-beta-propiothetin | Danggui |
| HBIN024060 | dimethyl camphorate | Danggui |
| HBIN024075 | dimethyl phthalate | Danggui |
| HBIN024083 | dimethyl sulfone | Danggui |
| HBIN024368 | dodecane | Danggui |
| HBIN024378 | dodecanol | Danggui |
| HBIN024386 | dodecenoicacid | Danggui |
| HBIN024390 | Dodekan | Danggui |
| HBIN025221 | (E)-octadec-3-ene | Danggui |
| HBIN025801 | ESEN | Danggui |
| HBIN025828 | ethanol | Danggui |
| HBIN025841 | Ethol | Danggui |
| HBIN025885 | ETHYLBENZALDEHYDE | Danggui |
| HBIN025973 | ethyl-p-methoxycinnamate | Danggui |
| HBIN026011 | eucalyptin | Danggui |
| HBIN026440 | FER | Danggui |
| HBIN026468 | FERULIC ACID (CIS) | Danggui |
| HBIN026629 | Folinic acid | Danggui |
| HBIN026630 | foliosidine | Danggui |
| HBIN027100 | gamma-acoradiene | Danggui |
| HBIN028447 | (-)-Guaia-1(10),11-dien-15-al | Danggui |
| HBIN028464 | guaiacol | Danggui |
| HBIN028520 | guanosine | Danggui |
| HBIN029095 | heptanal | Danggui |
| HBIN029443 | h-Met-h | Danggui |
| HBIN029511 | homosenkyunolide h | Danggui |
| HBIN029512 | homosenkyunolide i | Danggui |
| HBIN030079 | InChI=1/C15H24/c1-10-7-8-15-9-12(10)14(3,4)13(15)6-5-11(15)2/h7,11-13H,5-6,8-9H2,1-4H | Danggui |
| HBIN030413 | Isoamylbenzene | Danggui |
| HBIN030594 | isocnidilide | Danggui |
| HBIN030595 | isococculidine | Danggui |
| HBIN030728 | isoeugenol | Danggui |
| HBIN030734 | Isofernene | Danggui |
| HBIN031296 | Isotetandrine | Danggui |
| HBIN031367 | Isoxylaldehyde | Danggui |
| HBIN032801 | L-beta,gamma-Dimyristoyl-alpha-cephalin | Danggui |
| HBIN032833 | lecithin | Danggui |
| HBIN032850 | (+)-Ledol | Danggui |
| HBIN033036 | Levistolid A | Danggui |
| HBIN033198 | Ligustilide | Danggui |
| HBIN033201 | ligustilide dimer | Danggui |
| HBIN033241 | limocitrin-beta-d-glucoside | Danggui |
| HBIN033274 | linalyl acetate | Danggui |
| HBIN033580 | Loxanol V | Danggui |
| HBIN034524 | Maruzen M | Danggui |
| HBIN034596 | m-cresol | Danggui |
| HBIN034783 | Mesitaldehyde | Danggui |
| HBIN035120 | Methylbutenol | Danggui |
| HBIN035376 | m-Ethylphenol | Danggui |
| HBIN036309 | naphthalene1 | Danggui |
| HBIN036448 | n-butylidene phthalide | Danggui |
| HBIN037629 | o-Acetyl-p-cresol | Danggui |
| HBIN038486 | o-Xylenol | Danggui |
| HBIN039000 | PCR | Danggui |
| HBIN039001 | p-cresol | Danggui |
| HBIN039184 | pentylbenzene | Danggui |
| HBIN039362 | p-ethylphenol | Danggui |
| HBIN039500 | phenylacetic acid | Danggui |
| HBIN039613 | phosphatdic acid | Danggui |
| HBIN039618 | phosphatidylinositol | Danggui |
| HBIN039619 | phosphatidylinositol_qt | Danggui |
| HBIN039662 | phthalicanhydride | Danggui |
| HBIN039724 | Phyllanthin | Danggui |
| HBIN043548 | sebacicacid | Danggui |
| HBIN043549 | Sebiferic acid | Danggui |
| HBIN043556 | sec-butyl isothiocyanate | Danggui |
| HBIN043605 | sedanolide | Danggui |
| HBIN043723 | senkyunolide | Danggui |
| HBIN043727 | senkyunolide-C | Danggui |
| HBIN043728 | senkyunolide D | Danggui |
| HBIN043729 | senkyunolide E | Danggui |
| HBIN044506 | sphingomyelin | Danggui |
| HBIN044945 | Stigmasteryl ferulate | Danggui |
| HBIN045063 | succinic acid(high dose) | Danggui |
| HBIN045071 | suchilactone | Danggui |
| HBIN046494 | TMHYDROP | Danggui |
| HBIN047005 | tridecane | Danggui |
| HBIN047017 | Tridecylene | Danggui |
| HBIN047260 | Tropone | Danggui |
| HBIN047536 | Undecanol-6 | Danggui |
| HBIN047561 | uracil | Danggui |
| HBIN047579 | uridine | Danggui |
| HBIN047627 | Usaf hc-1 | Danggui |
| HBIN047719 | valerosidatum | Danggui |
| HBIN047744 | vanillin | Danggui |
| HBIN047745 | vanillin acetate | Danggui |
| HBIN048041 | vitamin b15 | Danggui |
| HBIN048349 | WLN: QR CQ DV1 | Danggui |
| HBIN048353 | WLN: QVR BVQ | Danggui |
| HBIN048740 | (Z)-2-Hexenyl hexanoate | Danggui |
| HBIN048749 | (Z)-2-[[(Z)-2-methylbut-2-enoyl]oxymethyl]but-2-enoic acid | Danggui |
| HBIN048763 | z-3',8',3'α,7'α-tetrahydro-6,3',7,7'α-diligustilide-8'-one | Danggui |
| HBIN049035 | z-ligustilide | Danggui |
| HBIN049099 | γ-acoradiene | Danggui |
| HBIN049180 | Δ2,4-dihydrophthalicanhydride | Danggui |
| HBIN049201 | δ-acoradiene | Danggui |
| HBIN042130 | retinol | Danggui, |
| HBIN000951 | 12-O-Nicotinoylisolineolone | Danggui, Dazao |
| HBIN021979 | curcumenol | Danggui, Dazao |
| HBIN021985 | curcumin | Danggui, Dazao |
| HBIN021990 | curcumol | Danggui, Dazao |
| HBIN021993 | curdione | Danggui, Dazao |
| HBIN022017 | curzerene | Danggui, Dazao |
| HBIN022018 | curzerenone | Danggui, Dazao |
| HBIN025326 | epicurcumenol | Danggui, Dazao |
| HBIN025327 | epicurzerenone | Danggui, Dazao |
| HBIN028358 | gqeieureulactene | Danggui, Dazao |
| HBIN030634 | isocurcumenol | Danggui, Dazao |
| HBIN033170 | lignoceric acid | Danggui, Dazao |
| HBIN036608 | neocurdione | Danggui, Dazao |
| HBIN036907 | nicotinic acid | Danggui, Dazao |
| HBIN043607 | sederone | Danggui, Dazao |
| HBIN044939 | Stigmasterol-beta-D-glucoside | Danggui, Dazao |
| HBIN048039 | vitamin b1 | Danggui, Dazao |
| HBIN048040 | vitamin b12 | Danggui, Dazao |
| HBIN018337 | beta-Terpinene | Danggui, Dazao |
| HBIN024079 | dimethyl sebacate | Danggui, Dazao |
| HBIN026465 | ferulic acid | Danggui, Dazao |
| HBIN043445 | Scopoletol | Danggui, Dazao |
| HBIN047563 | Uralene | Danggui, Dazao |
| HBIN048366 | WLN: VH6 | Danggui, Dazao |
| HBIN010878 | (4S)-1-methyl-4-(6-methylhepta-1,5-dien-2-yl)cyclohexene | Danggui, Dazao |
| HBIN018263 | β-selinene | Danggui, Dazao |
| HBIN019252 | cadinene | Danggui, Dazao |
| HBIN020253 | CHEBI:7 | Danggui, Dazao |
| HBIN028522 | Guasol | Danggui, Dazao |
| HBIN029328 | hexanoic acid | Danggui, Dazao |
| HBIN029840 | Hypnon | Danggui, Dazao |
| HBIN030257 | IPH | Danggui, Dazao |
| HBIN037213 | NON | Danggui, Dazao |
| HBIN037713 | o-cresol | Danggui, Dazao |
| HBIN038398 | o-Thymol | Danggui, Dazao |
| HBIN040754 | procurcumenol | Danggui, Guizhi, Dazao |
| HBIN020014 | cedrol | Danggui, Guizhi, Shaoyao |
| HBIN044152 | Sitogluside | Danggui, Guizhi, Shaoyao, Dazao |
| HBIN018278 | beta-sitosterol | Danggui, Guizhi, Shaoyao, Gancao, Dazao |
| HBIN036067 | myrcene | Danggui, Guizhi, Shaoyao, Xixin |
| HBIN036308 | naphthalene | Danggui, Guizhi, Shaoyao, Xixin |
| HBIN018094 | beta-elemene | Danggui, Guizhi, Shaoyao, Xixin, Dazao |
| HBIN015651 | (-)-alpha-Pinene | Danggui, Guizhi, Xixin |
| HBIN017987 | β-bisabolene | Danggui, Guizhi, Xixin |
| HBIN021904 | cuminal | Danggui, Guizhi, Xixin |
| HBIN022407 | Cymol | Danggui, Guizhi, Xixin |
| HBIN026379 | Farnesene | Danggui, Guizhi, Xixin |
| HBIN029013 | Hemo-sol | Danggui, Guizhi, Xixin |
| HBIN035798 | Moslene | Danggui, Guizhi, Xixin |
| HBIN042760 | Safrol | Danggui, Guizhi, Xixin |
| HBIN038680 | palmitic acid | Danggui, Guizhi, Xixin, Dazao |
| HBIN002121 | 1,8-dimethyl-4-(1-methylenyl)-spiro(4,5)-dec-7-ene | Danggui, Shaoyao |
| HBIN003338 | 1-tetradecanol | Danggui, Shaoyao |
| HBIN009381 | 3-o-tetradecanoyl-1-cyano-2-methyl-1,2-propene | Danggui, Shaoyao |
| HBIN016087 | angelicin | Danggui, Shaoyao |
| HBIN018233 | beta-phellandrene | Danggui, Shaoyao |
| HBIN030819 | isoimperatorin | Danggui, Shaoyao |
| HBIN039427 | phellatin | Danggui, Shaoyao |
| HBIN039478 | phenol | Danggui, Shaoyao |
| HBIN043446 | scopolin | Danggui, Shaoyao |
| HBIN003405 | 20-Hexadecanoylingenol | Danggui, Shaoyao, Dazao |
| HBIN036159 | myristic acid | Danggui, Shaoyao, Dazao |
| HBIN043442 | scopoletin | Danggui, Shaoyao, Dazao |
| HBIN019487 | camphene | Danggui, Shaoyao, Xixin |
| HBIN033245 | limonene | Danggui, Shaoyao, Xixin |
| HBIN015652 | alpha-pinene | Danggui, Shaoyao, Xixin, Dazao |
| HBIN029268 | hexadecanoic acid | Danggui, Shaoyao, Xixin, Dazao |
| HBIN044918 | stigmasterol | Danggui, Tongcao, Dazao |
| HBIN008383 | 3-carene | Danggui, Xixin |
| HBIN015702 | ()-alpha-Terpineol | Danggui, Xixin |
| HBIN015704 | alpha-terpineol | Danggui, Xixin |
| HBIN018019 | beta-Chamigrene | Danggui, Xixin |
| HBIN019493 | Campherenol | Danggui, Xixin |
| HBIN019786 | carvacrol | Danggui, Xixin |
| HBIN019788 | carvacrol acetate | Danggui, Xixin |
| HBIN020955 | cis-Thujopsene | Danggui, Xixin |
| HBIN021921 | ()-Cuparene | Danggui, Xixin |
| HBIN024384 | DODECENE | Danggui, Xixin |
| HBIN026019 | Eucarvone | Danggui, Xixin |
| HBIN037250 | nonanal | Danggui, Xixin |
| HBIN040347 | p-Ocimene | Danggui, Xixin |
| HBIN045062 | succinic acid | Danggui, Xixin |
| HBIN046033 | tetradecane | Danggui, Xixin |
| HBIN036162 | myristicin | Danggui, Xixin, Dazao |
| HBIN001396 | 1-(4-Coumaroyl)alpha-rhamnopyranose | Dazao |
| HBIN002080 | 18658-41-8 | Dazao |
| HBIN002852 | 1-O-β-D-glucopyranosyl-(2S,3S,4R,8E)-2-[(2′R)-2'-hydroxy-tetracosanoyl]-8-octadecene-1,3,4-triol | Dazao |
| HBIN002952 | 1-(p-coumaroyl)-alpha-l-rhamnopyranose | Dazao |
| HBIN003203 | (1S,2R,4aS,6aR,6aS,6bR,8aR,12aR,14bS)-1,2,6a,6b,9,9,12a-heptamethyl-10-oxo-1,2,3,4,5,6,6a,7,8,8a,11,12,13,14b-tetradecahydropicene-4a-carboxylic acid | Dazao |
| HBIN003333 | 1-terpinen-4-ol | Dazao |
| HBIN003526 | 21302-79-4 | Dazao |
| HBIN004305 | 24-Dehydrocholesterol | Dazao |
| HBIN005231 | 2α,3β-dihydroxyolean-12-en-28-oicacid | Dazao |
| HBIN005294 | 2α-hydroxyoleanolic acid | Dazao |
| HBIN005301 | 2α-hydroxyursolic acid | Dazao |
| HBIN006269 | (2-O-trans-p-coumaroyl-alphitolic acid | Dazao |
| HBIN006713 | (2S,3S,4R,8E)-2-(2' R)-2'-hydroxy-tetracosanoyl-8-octadecene-1,3,4-triol］ | Dazao |
| HBIN006714 | (2S,3S,4R,8E)-2-[(2′R)-2′-hydroxy-tetracosanoyl]-8-octadecene-1,3,4-triol | Dazao |
| HBIN007058 | 3-[[(2S)-2,4-dihydroxy-3,3-dimethylbutanoyl]amino]propanoic acid | Dazao |
| HBIN008110 | 3β,6β-stigmast-4-en-3,6-diol | Dazao |
| HBIN009292 | 3-o-(cis-p-coumaroyl)-alphitolicacid | Dazao |
| HBIN009293 | 3-O-cis-p-coumaroyl alphitolic acid | Dazao |
| HBIN009294 | 3-o-(cis-p-coumaroyl)-maslinicacid | Dazao |
| HBIN009295 | 3-O-cis-p-coumaroyl-maslinic acid | Dazao |
| HBIN009387 | 3-o-(trans-p-coumaroyl)-alphitolicacid | Dazao |
| HBIN009388 | 3-O-trans-p-coumaroyl-alphitolic acid | Dazao |
| HBIN009389 | 3-o-(trans-p-coumaroyl)-maslinicacid | Dazao |
| HBIN009430 | 3-oxo-olean-12-en-28-oicacid | Dazao |
| HBIN009687 | (3S,6R,8S,9S,10R,13R,14S,17R)-17-[(1R,4R)-4-ethyl-1,5-dimethylhexyl]-10,13-dimethyl-2,3,6,7,8,9,11,12,14,15,16,17-dodecahydro-1H-cyclopenta[a]phenanthrene-3,6-diol | Dazao |
| HBIN010476 | 4-hydroxy-3-methoxy benzoic acid | Dazao |
| HBIN010856 | (4R)-4-hydroxy-4-[(E,3S)-3-hydroxybut-1-enyl]-3,5,5-trimethylcyclohex-2-en-1-one | Dazao |
| HBIN010884 | (4S)-4-hydroxy-3,5,5-trimethyl-4-[(E,3R)-3-[(2R,3R,4S,5S,6R)-3,4,5-trihydroxy-6-(hydroxymethyl)tetrahydropyran-2-yl]oxybut-1-enyl]cyclohex-2-en-1-one | Dazao |
| HBIN010887 | (4S)-4-hydroxy-4-[(E,3S)-3-hydroxybut-1-enyl]-3,5,5-trimethylcyclohex-2-en-1-one | Dazao |
| HBIN012112 | 6,8-di-C-glucsoyl-2(R)-naringenin | Dazao |
| HBIN012113 | 6,8-di-C-glucsoyl-2(s)-naringenin | Dazao |
| HBIN012343 | 6'''-feruloylspinosin | Dazao |
| HBIN012709 | 6'-O-p-Coumaroylgenipingentiobioside | Dazao |
| HBIN012710 | 6''-o-p-coumaroylgenipingentiobioside | Dazao |
| HBIN012752 | 6'''-p-coumaroylspinosin | Dazao |
| HBIN012822 | 6'''-sinapoylspinosin | Dazao |
| HBIN014667 | acylatedflavuone-C-glycoside Ⅰ | Dazao |
| HBIN014668 | ADA | Dazao |
| HBIN014735 | Adouetine X | Dazao |
| HBIN015749 | alphitolic acid | Dazao |
| HBIN015750 | alphitotic acid | Dazao |
| HBIN015864 | amiphibine H | Dazao |
| HBIN017056 | ASI | Dazao |
| HBIN017075 | Asimilobine | Dazao |
| HBIN017893 | berberine | Dazao |
| HBIN018006 | beta carotene | Dazao |
| HBIN018374 | betulin | Dazao |
| HBIN018379 | betulinic acid | Dazao |
| HBIN018382 | Betulonic acid | Dazao |
| HBIN019933 | (+)-Catechin-5-O-glucoside | Dazao |
| HBIN019948 | catechol | Dazao |
| HBIN019953 | Catharanthamine | Dazao |
| HBIN019994 | ceanothenic acid | Dazao |
| HBIN019995 | ceanothic acid | Dazao |
| HBIN020796 | cis-9,cis-12-linoleicacid | Dazao |
| HBIN021159 | CMP | Dazao |
| HBIN021288 | colubrinicacid | Dazao |
| HBIN021620 | coumestrol | Dazao |
| HBIN021802 | Crystal VI | Dazao |
| HBIN022543 | Daechualkaloid A | Dazao |
| HBIN022544 | daechualkoloid A | Dazao |
| HBIN022545 | daechucyclopride Ⅰ | Dazao |
| HBIN022546 | Daechuine S10 | Dazao |
| HBIN022547 | Daechuine S26 | Dazao |
| HBIN022548 | daechuine S3 | Dazao |
| HBIN022549 | Daechuine S5 | Dazao |
| HBIN022550 | Daechuine S6 | Dazao |
| HBIN022551 | Daechuine S7 | Dazao |
| HBIN022552 | Daechuine S8-1 | Dazao |
| HBIN022553 | daechunine S1 | Dazao |
| HBIN022554 | daechunine S10 | Dazao |
| HBIN022555 | daechunine S2 | Dazao |
| HBIN022556 | daechunine S26 | Dazao |
| HBIN022557 | daechunine S27 | Dazao |
| HBIN022558 | daechunine S3 | Dazao |
| HBIN022559 | daechunine S4 | Dazao |
| HBIN022560 | daechunine S5 | Dazao |
| HBIN022561 | daechunine S6 | Dazao |
| HBIN022562 | daechunine S7 | Dazao |
| HBIN022563 | daechunine S8-1 | Dazao |
| HBIN022564 | daechunine S9 | Dazao |
| HBIN022705 | darutoside | Dazao |
| HBIN022755 | Daturic acid | Dazao |
| HBIN022771 | daucosterol | Dazao |
| HBIN023530 | D-glucose | Dazao |
| HBIN023807 | dihydroalpitolic acid methyl ester | Dazao |
| HBIN025318 | epiceanothic acid | Dazao |
| HBIN026829 | Fumarine | Dazao |
| HBIN028044 | Glucosol | Dazao |
| HBIN028076 | GLY | Dazao |
| HBIN029081 | heptadecanoic acid | Dazao |
| HBIN029980 | IES | Dazao |
| HBIN031565 | jubanine A | Dazao |
| HBIN031566 | Jubanine-A | Dazao |
| HBIN031567 | jubanine B | Dazao |
| HBIN031568 | jubanine C | Dazao |
| HBIN031569 | jubanine D | Dazao |
| HBIN031570 | jubasaponin Ⅰ | Dazao |
| HBIN031585 | jujubasaponim Ⅰ | Dazao |
| HBIN031586 | jujubasaponin IV | Dazao |
| HBIN031587 | jujubasaponin IV_qt | Dazao |
| HBIN031588 | Jujubasaponin V | Dazao |
| HBIN031589 | Jujubasaponin VI | Dazao |
| HBIN031590 | Jujubasaponin VI_qt | Dazao |
| HBIN031591 | Jujubasaponin V_qt | Dazao |
| HBIN031592 | Jujubogenin | Dazao |
| HBIN031594 | Jujuboside | Dazao |
| HBIN031595 | jujuboside a | Dazao |
| HBIN031596 | Jujuboside A1 | Dazao |
| HBIN031597 | jujuboside A_qt | Dazao |
| HBIN031598 | jujuboside b | Dazao |
| HBIN031599 | jujuboside b1 | Dazao |
| HBIN031601 | Jujuboside C | Dazao |
| HBIN031602 | Jujuboside C_qt | Dazao |
| HBIN032992 | Leucinum | Dazao |
| HBIN033330 | Linoleic | Dazao |
| HBIN033368 | Liquidambaric acid | Dazao |
| HBIN033583 | LPG | Dazao |
| HBIN033751 | lupeol | Dazao |
| HBIN033890 | L-Valin | Dazao |
| HBIN034033 | Lysicamine | Dazao |
| HBIN034310 | malic acid | Dazao |
| HBIN034311 | malkangunin | Dazao |
| HBIN034527 | Maslinic acid | Dazao |
| HBIN034566 | Mauritine A | Dazao |
| HBIN034568 | Mauritine D | Dazao |
| HBIN034833 | Methose | Dazao |
| HBIN035598 | MLT | Dazao |
| HBIN035808 | Moupinamide | Dazao |
| HBIN037170 | N-nornuciforine | Dazao |
| HBIN037367 | Nornuciferine | Dazao |
| HBIN037567 | Nuciferin | Dazao |
| HBIN037588 | nummularine A | Dazao |
| HBIN038023 | oleanonic acid | Dazao |
| HBIN038993 | PCG | Dazao |
| HBIN040523 | Pomolic acid | Dazao |
| HBIN040811 | Prolinum | Dazao |
| HBIN040936 | protopine | Dazao |
| HBIN040937 | Protoporphyrin | Dazao |
| HBIN042307 | riboflavine | Dazao |
| HBIN042420 | roseoside | Dazao |
| HBIN042675 | Ruvoside_qt | Dazao |
| HBIN042680 | (r)-zizyphusine | Dazao |
| HBIN043428 | (S)-Coclaurine | Dazao |
| HBIN043539 | Scutianine C | Dazao |
| HBIN044070 | Sinapyl alcohol | Dazao |
| HBIN044525 | spinosin | Dazao |
| HBIN044527 | spinosin_qt | Dazao |
| HBIN044531 | Spiradine A | Dazao |
| HBIN044735 | stearin | Dazao |
| HBIN044797 | stepharine | Dazao |
| HBIN044799 | Stepholidine | Dazao |
| HBIN045194 | swertisin | Dazao |
| HBIN045205 | Sylvestrene | Dazao |
| HBIN045485 | tannin | Dazao |
| HBIN045516 | Tar | Dazao |
| HBIN045552 | tartaric acid | Dazao |
| HBIN045579 | tauremisin | Dazao |
| HBIN046021 | tetracosanoic acid | Dazao |
| HBIN046150 | TGL | Dazao |
| HBIN046299 | Thiamine | Dazao |
| HBIN046705 | trans-9-trans-12-linoleic acid | Dazao |
| HBIN047135 | trimyristin | Dazao |
| HBIN047137 | triolein | Dazao |
| HBIN047242 | Trochol | Dazao |
| HBIN047555 | Uosolic Acid | Dazao |
| HBIN047621 | ursonic acid | Dazao |
| HBIN047679 | Vaccenic acid | Dazao |
| HBIN048044 | vitamin b5 | Dazao |
| HBIN048066 | Vitamin- G | Dazao |
| HBIN048166 | Vomifoliol | Dazao |
| HBIN048174 | Vulgarin | Dazao |
| HBIN048342 | WLN: Q1R | Dazao |
| HBIN049012 | Ziziphin | Dazao |
| HBIN049013 | Ziziphin_qt | Dazao |
| HBIN049014 | ziziphussaponin i | Dazao |
| HBIN049015 | ziziphussaponin ii | Dazao |
| HBIN049016 | ziziphussaponin iii | Dazao |
| HBIN049017 | Zizybeoside Ⅱ | Dazao |
| HBIN049018 | Zizybeoside I | Dazao |
| HBIN049019 | zizybeoside II | Dazao |
| HBIN049020 | zizyberanal acid | Dazao |
| HBIN049021 | zizyberanalic acid | Dazao |
| HBIN049022 | zizyberenalicacid | Dazao |
| HBIN049023 | Zizyphine A | Dazao |
| HBIN049025 | zizyphus saponin I | Dazao |
| HBIN049026 | Zizyphus saponin II | Dazao |
| HBIN049027 | Zizyphus saponin III | Dazao |
| HBIN049028 | zizyphus saponin I_qt | Dazao |
| HBIN049029 | zizyvoside Ⅰ | Dazao |
| HBIN049030 | zizyvoside I | Dazao |
| HBIN049031 | zizyvoside II | Dazao |
| HBIN049051 | zoomaric acid | Dazao |
| HBIN049068 | Zuztvisude I | Dazao |
| HBIN049168 | γ-terpinene | Dazao |
| HBIN000391 | 11-deoxyglycyrrhetic acid | Gancao |
| HBIN000905 | 12-methyltetradecanoate | Gancao |
| HBIN001196 | 1,3-dihydroxy-8,9-dimethoxy-6-benzofurano[3,2-c]chromenone | Gancao |
| HBIN001197 | 1,3-dihydroxy-9-methoxy-6-benzofurano[3,2-c]chromenone | Gancao |
| HBIN001683 | 1-(5-hydroxy-2,2-dimethylchromen-6-yl)-3-(4-hydroxyphenyl)prop-2-en-1-one | Gancao |
| HBIN002087 | 18alpha-glycyrrhetinic acid | Gancao |
| HBIN002089 | 18α-hydroxyglycyrrhetic acid | Gancao |
| HBIN002095 | 18beta-glycyrrhetinic acid | Gancao |
| HBIN002713 | 1-Methoxyficifolinol | Gancao |
| HBIN002717 | 1-Methoxyphaseollidin | Gancao |
| HBIN003221 | (1S,2S)-1,2-dimethylcyclopentane | Gancao |
| HBIN003537 | 21987_FLUKA | Gancao |
| HBIN003683 | 22β-acetylglabric acid | Gancao |
| HBIN003712 | 2,2-DIMETHYLPENTANE | Gancao |
| HBIN003876 | 2-(3,4-dihydroxyphenyl)-5,7-dihydroxy-6-(3-methylbut-2-enyl)chromone | Gancao |
| HBIN004066 | 2,3-dimethylhexane | Gancao |
| HBIN004155 | 2-[(3R)-8,8-dimethyl-3,4-dihydro-2H-pyrano[6,5-f]chromen-3-yl]-5-methoxyphenol | Gancao |
| HBIN004213 | 2,4,4'-trihydroxychalcone | Gancao |
| HBIN004397 | 24-Hydroxy-11-deoxyglycyrrhetic acid | Gancao |
| HBIN004409 | 24-Hydroxyglycyrrhetic acid | Gancao |
| HBIN004636 | 2,5-dihydroxymethyl-3,4-dihydroxypyrrolidine | Gancao |
| HBIN004827 | 2,6,10-trimethyl-dodecane | Gancao |
| HBIN005018 | 2',7-Dihydroxy-4'-methoxyisoflavan-7-O-β-d-glucopyranoside | Gancao |
| HBIN005450 | 2-Caren-10-al | Gancao |
| HBIN005625 | 2-Ethyl-p-xylene | Gancao |
| HBIN005662 | 2-heptanone | Gancao |
| HBIN005940 | 2-Methyl-1,3,6-trihydroxyanthraquinone | Gancao |
| HBIN006004 | 2-methyl-5-propyl nonane | Gancao |
| HBIN006008 | 2-methyl-6-ethyl decane | Gancao |
| HBIN006356 | (2R)-1-[2,4-dihydroxy-5-(3-methylbut-2-enyl)phenyl]-2-hydroxy-3-[4-hydroxy-3-(3-methylbut-2-enyl)phenyl]propan-1-one | Gancao |
| HBIN006363 | (2R)-2-[3,4-dihydroxy-5-(3-methylbut-2-enyl)phenyl]-5,7-dihydroxy-8-(3-methylbut-2-enyl)chroman-4-one | Gancao |
| HBIN006533 | (2R)-7-hydroxy-2-[4-hydroxy-3-(3-methylbut-2-enyl)phenyl]chroman-4-one | Gancao |
| HBIN006534 | (2R)-7-hydroxy-2-(4-hydroxyphenyl)chroman-4-one | Gancao |
| HBIN006585 | (2S)-2-[4-hydroxy-3-(3-methylbut-2-enyl)phenyl]-8,8-dimethyl-2,3-dihydropyrano[2,3-f]chromen-4-one | Gancao |
| HBIN006773 | (2S)-6-(2,4-dihydroxyphenyl)-2-(2-hydroxypropan-2-yl)-4-methoxy-2,3-dihydrofuro[3,2-g]chromen-7-one | Gancao |
| HBIN006789 | (2S)-7-hydroxy-2-(4-hydroxyphenyl)-8-(3-methylbut-2-enyl)chroman-4-one | Gancao |
| HBIN006833 | 2-Tetradecanone | Gancao |
| HBIN006998 | 3,22-Dihydroxy-11-oxo-delta(12)-oleanene-27-alpha-methoxycarbonyl-29-oic acid | Gancao |
| HBIN007021 | 3-(2,4-dihydroxyphenyl)-8-(1,1-dimethylprop-2-enyl)-7-hydroxy-5-methoxy-coumarin | Gancao |
| HBIN007042 | 3-(2-hydroxy-4-methoxyphenyl)-2H-chromen-7-ol | Gancao |
| HBIN007125 | 3-(3,4-dihydroxyphenyl)-5,7-dihydroxy-8-(3-methylbut-2-enyl)chromone | Gancao |
| HBIN007191 | 3,3-Dimethylpentane | Gancao |
| HBIN007192 | 3,3'-dimethylquercetin | Gancao |
| HBIN007212 | 3,4,3',4'-Tetrahydroxy-2-methoxychalcone | Gancao |
| HBIN007313 | 3-[4,6-dihydroxy-2-methoxy-3-(3-methylbut-2-enyl)phenyl]-7-hydroxy-chromone | Gancao |
| HBIN007332 | 3,4-dicaffeoyl-5-(3-hydroxy-3-methyl) glutaroyl quinic acid | Gancao |
| HBIN008213 | 3β-formylglabrolide | Gancao |
| HBIN008534 | 3-Ethylpentane | Gancao |
| HBIN008674 | 3'-Hydroxy-4'-O-Methylglabridin | Gancao |
| HBIN008713 | 3-Hydroxyglabrol | Gancao |
| HBIN008715 | 3-hydroxyglabrol (ii) | Gancao |
| HBIN008831 | 3'-Methoxyglabridin | Gancao |
| HBIN008897 | 3-methyl-6,7,8-trihydropyrrolo[1,2-a]pyrimidin-2-one | Gancao |
| HBIN008944 | 3-methylheptane | Gancao |
| HBIN008949 | 3-methylhexane | Gancao |
| HBIN008962 | 3-Methylpentane | Gancao |
| HBIN009025 | 3-o-acetyl-glycyrrhetinicacid | Gancao |
| HBIN009235 | 3-o-[β-d-glucuronopyranosyl-(1→2)-o-β-d-glucuronopyranosyl]-24-hydroxyglabrolide | Gancao |
| HBIN009594 | (3S)-2,3-dimethylpentane | Gancao |
| HBIN009773 | 3'-(γ,γ-dimethylallyl)-kievitone | Gancao |
| HBIN009854 | 4,2',4',alpha-Tetrahydroxydihydrochalcone | Gancao |
| HBIN010407 | 4H-1-Benzopyran-4-one, 2-(4-(beta-D-glucopyranosyloxy)phenyl)-2,3-dihydro-5,7-dihydroxy-, (2S)- | Gancao |
| HBIN010785 | 4'-o-methylglabridin | Gancao |
| HBIN010879 | (4S)-2,4-dimethylhexane | Gancao |
| HBIN011105 | 5,6,7,8-Tetrahydro-2,4-dimethylquinoline | Gancao |
| HBIN011106 | 5,6,7,8-Tetrahydro-4-methylquinoline | Gancao |
| HBIN011272 | 5,7-dihydroxy-3-(2-hydroxy-4-methoxy-phenyl)-6-(3-methylbut-2-enyl)chromone | Gancao |
| HBIN011278 | 5,7-dihydroxy-3-(4-methoxyphenyl)-8-(3-methylbut-2-enyl)chromone | Gancao |
| HBIN012104 | 6,8-bis(c-β-glucosyl)-apigenin | Gancao |
| HBIN012604 | 6″-O-acetylliquiritin | Gancao |
| HBIN012759 | 6-prenylated eriodictyol | Gancao |
| HBIN012887 | 7,2',4'-trihydroxy－5-methoxy-3－arylcoumarin | Gancao |
| HBIN012928 | 7,4'-Dihydroxyflavone | Gancao |
| HBIN013015 | 7-Acetoxy-2-methylisoflavone | Gancao |
| HBIN013209 | 7-hydroxy-2-[4-hydroxy-3-(3-methylbut-2-enyl)phenyl]-6-(3-methylbut-2-enyl)chromone | Gancao |
| HBIN013215 | 7-hydroxy-2-methyl-3-phenyl-chromone | Gancao |
| HBIN013310 | 7-Methoxy-2-methyl isoflavone | Gancao |
| HBIN013572 | 8-(6-hydroxy-2-benzofuranyl)-2,2-dimethyl-5-chromenol | Gancao |
| HBIN013807 | 8-methoxy-5-o-glucoside flavone | Gancao |
| HBIN013823 | 8-methyl-10-hydroxylycoctonine | Gancao |
| HBIN013880 | 8-prenylated eriodictyol | Gancao |
| HBIN013888 | 8-Prenylwighteone | Gancao |
| HBIN015737 | alpha-trihydroxy coprostanic acid | Gancao |
| HBIN015954 | amylum | Gancao |
| HBIN016489 | apioglycyrrhizin | Gancao |
| HBIN016490 | apioglycyrrhizin_qt | Gancao |
| HBIN016566 | Araboglycyrrhizin | Gancao |
| HBIN016567 | Araboglycyrrhizin_qt | Gancao |
| HBIN016568 | Arachic acid | Gancao |
| HBIN016994 | Artonin E | Gancao |
| HBIN017931 | berniarin | Gancao |
| HBIN018136 | beta-Glycyrrhetinic acid | Gancao |
| HBIN019035 | BuOH | Gancao |
| HBIN019111 | butylated hydroxytoluene | Gancao |
| HBIN019425 | calycosin | Gancao |
| HBIN019894 | Castanin | Gancao |
| HBIN021558 | Corylifolinin | Gancao |
| HBIN022166 | Cyclobutanol, 1-ethyl- | Gancao |
| HBIN022581 | Daidzein dimethyl ether | Gancao |
| HBIN023041 | dehydroglyasperins C | Gancao |
| HBIN023519 | DFV | Gancao |
| HBIN023635 | dibutyl uralsaponin a ester | Gancao |
| HBIN024361 | Docosyl caffeate | Gancao |
| HBIN024593 | (e)-1-[2,4-dihydroxy-3-(3-methyl-2-butenyl)phenyl]-3-(2,2-dimethyl-8-hydroxy-2h-benzo-pyran-6-yl)-2-propen-1-one | Gancao |
| HBIN024594 | (e)-1-[2,4-dihydroxy-3-(3-methyl-2-butenyl)phenyl]-3-(4-hydroxy-3-[3-methyl-2-butenyl)phenyl]-2-propen-1-one | Gancao |
| HBIN024595 | (E)-1-[2,4-dihydroxy-3-(3-methylbut-2-enyl)phenyl]-3-(2,4-dihydroxyphenyl)prop-2-en-1-one | Gancao |
| HBIN024596 | (E)-1-[2,4-dihydroxy-3-(3-methylbut-2-enyl)phenyl]-3-[4-hydroxy-3-(3-methylbut-2-enyl)phenyl]prop-2-en-1-one | Gancao |
| HBIN024597 | (E)-1-(2,4-dihydroxyphenyl)-3-(2,2-dimethylchromen-6-yl)prop-2-en-1-one | Gancao |
| HBIN024598 | (E)-1-(2,4-dihydroxyphenyl)-3-[4-hydroxy-3-(3-methylbut-2-enyl)phenyl]prop-2-en-1-one | Gancao |
| HBIN024617 | (E)-1-butoxyhex-2-ene | Gancao |
| HBIN024662 | (E)-3-[3,4-dihydroxy-5-(3-methylbut-2-enyl)phenyl]-1-(2,4-dihydroxyphenyl)prop-2-en-1-one | Gancao |
| HBIN024749 | EB | Gancao |
| HBIN024796 | echinatin | Gancao |
| HBIN024853 | (E)-dodec-2-ene | Gancao |
| HBIN025952 | ethyl-n-buthy-uralsaponin a esters | Gancao |
| HBIN026022 | euchrenone | Gancao |
| HBIN026243 | Eurycarpin A | Gancao |
| HBIN026662 | formononetin | Gancao |
| HBIN026663 | formononetin-7-glucoside | Gancao |
| HBIN026977 | gadelaidic acid | Gancao |
| HBIN027172 | Gancaonin A | Gancao |
| HBIN027173 | Gancaonin B | Gancao |
| HBIN027174 | Gancaonin C | Gancao |
| HBIN027175 | Gancaonin D | Gancao |
| HBIN027176 | gancaonin e | Gancao |
| HBIN027177 | gancaonin f | Gancao |
| HBIN027178 | Gancaonin G | Gancao |
| HBIN027179 | Gancaonin H | Gancao |
| HBIN027180 | Gancaonin I | Gancao |
| HBIN027185 | Gancaonin P | Gancao |
| HBIN027186 | gancaonin p-3'-methylether | Gancao |
| HBIN027187 | Gancaonin Q | Gancao |
| HBIN027188 | Gancaonin R | Gancao |
| HBIN027189 | Gancaonin S | Gancao |
| HBIN027190 | gancaonin T | Gancao |
| HBIN027191 | Gancaonin U | Gancao |
| HBIN027192 | Gancaonin V | Gancao |
| HBIN027193 | gancaonin x | Gancao |
| HBIN027226 | ganoderic acid A | Gancao |
| HBIN027842 | Glabranin | Gancao |
| HBIN027847 | Glabrene | Gancao |
| HBIN027849 | Glabridin | Gancao |
| HBIN027855 | glabrol | Gancao |
| HBIN027856 | glabrolide | Gancao |
| HBIN027857 | Glabrone | Gancao |
| HBIN027957 | Glepidotin A | Gancao |
| HBIN027958 | Glepidotin B | Gancao |
| HBIN027962 | glisoflavanone | Gancao |
| HBIN027988 | gloeosteretriol | Gancao |
| HBIN028048 | glucuronic acid | Gancao |
| HBIN028077 | glyarallin b | Gancao |
| HBIN028078 | Glyasperin A | Gancao |
| HBIN028079 | glyasperin B | Gancao |
| HBIN028080 | Glyasperin C | Gancao |
| HBIN028082 | glyasperin E | Gancao |
| HBIN028083 | glyasperin F | Gancao |
| HBIN028086 | glyasperins D | Gancao |
| HBIN028087 | Glyasperins K | Gancao |
| HBIN028088 | Glyasperins M | Gancao |
| HBIN028089 | glyasperins Z | Gancao |
| HBIN028174 | Glycycoumarin | Gancao |
| HBIN028176 | glycyphyllin | Gancao |
| HBIN028177 | Glycyram | Gancao |
| HBIN028180 | Glycyrin | Gancao |
| HBIN028181 | Glycyrol | Gancao |
| HBIN028182 | glycyroside | Gancao |
| HBIN028184 | glycyrrhetinic acid | Gancao |
| HBIN028185 | glycyrrhetol | Gancao |
| HBIN028186 | glycyrrhisoflavanone | Gancao |
| HBIN028187 | glycyrrhisoflavone | Gancao |
| HBIN028188 | Glycyrrhiza flavonol A | Gancao |
| HBIN028189 | glycyrrhiza-flavonol a | Gancao |
| HBIN028190 | glycyrrhizic acid | Gancao |
| HBIN028191 | glycyrrhizin | Gancao |
| HBIN028193 | glyeurysaponin | Gancao |
| HBIN028195 | glyinflanin A | Gancao |
| HBIN028197 | Glypallichalcone | Gancao |
| HBIN028202 | glyurallin a | Gancao |
| HBIN028204 | glyuranolide | Gancao |
| HBIN028208 | glyyunnanprosapogenin d | Gancao |
| HBIN028214 | Glyzaglabrin | Gancao |
| HBIN028215 | gmelofuran | Gancao |
| HBIN029092 | Heptan | Gancao |
| HBIN029234 | HEX | Gancao |
| HBIN029419 | Hirsutrin | Gancao |
| HBIN029422 | Hispaglabridin A | Gancao |
| HBIN029423 | Hispaglabridin B | Gancao |
| HBIN029430 | hispidulin | Gancao |
| HBIN029445 | HMO | Gancao |
| HBIN029973 | ICO | Gancao |
| HBIN029974 | icos-5-enoic acid | Gancao |
| HBIN030134 | Inermine | Gancao |
| HBIN030137 | Inflacoumarin A | Gancao |
| HBIN030738 | isoflavone | Gancao |
| HBIN030769 | isoglabrolide | Gancao |
| HBIN030772 | isoglycycoumarin | Gancao |
| HBIN030773 | Isoglycyrol | Gancao |
| HBIN030780 | isogosferol | Gancao |
| HBIN030782 | isograbrol | Gancao |
| HBIN030800 | ISOHEPTANE | Gancao |
| HBIN030802 | Isohexane | Gancao |
| HBIN030868 | Isolicoflavonol | Gancao |
| HBIN030869 | isoliensinine | Gancao |
| HBIN030895 | isoliquiriligenin | Gancao |
| HBIN030896 | isoliquiritigenin | Gancao |
| HBIN030898 | isoliquiritin | Gancao |
| HBIN030899 | isolobelanine | Gancao |
| HBIN031000 | Isoononin | Gancao |
| HBIN031002 | isoorientin | Gancao |
| HBIN031102 | isoquercitrin | Gancao |
| HBIN031109 | Isoramanone | Gancao |
| HBIN031114 | isorhamnetin | Gancao |
| HBIN031221 | isoschaftoside | Gancao |
| HBIN031319 | Isotrifoliol | Gancao |
| HBIN031320 | isotrilobine | Gancao |
| HBIN031345 | Isoviolanthin | Gancao |
| HBIN031404 | Izoforon | Gancao |
| HBIN031446 | Jaranol | Gancao |
| HBIN032060 | Kanzonol E | Gancao |
| HBIN032061 | Kanzonol F | Gancao |
| HBIN032063 | Kanzonol H | Gancao |
| HBIN032066 | kanzonol k | Gancao |
| HBIN032067 | kanzonol l | Gancao |
| HBIN032073 | kanzonols K | Gancao |
| HBIN032074 | kanzonols L | Gancao |
| HBIN032075 | kanzonols T | Gancao |
| HBIN032076 | kanzonols W | Gancao |
| HBIN032077 | kanzonols X | Gancao |
| HBIN032082 | Kanzonol Z | Gancao |
| HBIN032101 | Karenzu DK2 | Gancao |
| HBIN032874 | lensinine | Gancao |
| HBIN033076 | Licoagrocarpin | Gancao |
| HBIN033083 | Licoagroisoflavone | Gancao |
| HBIN033085 | licoagropin | Gancao |
| HBIN033090 | licobenzofuran | Gancao |
| HBIN033091 | licobichalcone | Gancao |
| HBIN033093 | licochalcone a | Gancao |
| HBIN033094 | Licochalcone B | Gancao |
| HBIN033095 | licochalcone C | Gancao |
| HBIN033096 | licochalconeD | Gancao |
| HBIN033098 | licochalcone G | Gancao |
| HBIN033099 | Licocoumarone | Gancao |
| HBIN033101 | Licoflavone | Gancao |
| HBIN033105 | Licoflavonol | Gancao |
| HBIN033106 | licofuranocoumarin | Gancao |
| HBIN033107 | licoisoflavanone | Gancao |
| HBIN033108 | licoisoflavaone | Gancao |
| HBIN033109 | Licoisoflavone | Gancao |
| HBIN033110 | Licoisoflavone B | Gancao |
| HBIN033111 | licoleafol | Gancao |
| HBIN033112 | Liconeolignan | Gancao |
| HBIN033114 | licopyranocoumarin | Gancao |
| HBIN033115 | Licorice glycoside A | Gancao |
| HBIN033121 | licorice glycoside E | Gancao |
| HBIN033124 | licoricesaponin a3 | Gancao |
| HBIN033125 | licoricesaponin b2 | Gancao |
| HBIN033126 | licorice-saponin B2 | Gancao |
| HBIN033127 | licoricesaponin c2 | Gancao |
| HBIN033128 | licorice-saponin C2 | Gancao |
| HBIN033129 | licorice-saponin C2_qt | Gancao |
| HBIN033130 | licoricesaponin d3 | Gancao |
| HBIN033131 | licoricesaponin e2 | Gancao |
| HBIN033132 | licoricesaponine a3 | Gancao |
| HBIN033134 | licoricesaponine c2 | Gancao |
| HBIN033135 | licoricesaponine d3 | Gancao |
| HBIN033136 | licoricesaponine f3 | Gancao |
| HBIN033137 | licoricesaponine g2 | Gancao |
| HBIN033138 | licoricesaponine h2 | Gancao |
| HBIN033139 | licoricesaponine j2 | Gancao |
| HBIN033140 | licoricesaponine k2 | Gancao |
| HBIN033141 | licoricesaponin f3 | Gancao |
| HBIN033142 | licorice-saponin F3 | Gancao |
| HBIN033143 | licorice-saponin F3_qt | Gancao |
| HBIN033144 | licoricesaponin g2 | Gancao |
| HBIN033145 | licorice-saponin G2 | Gancao |
| HBIN033146 | licorice-saponin G2_qt | Gancao |
| HBIN033147 | licoricesaponinh2 | Gancao |
| HBIN033148 | licorice-saponin H2 | Gancao |
| HBIN033149 | licorice-saponin H2_qt | Gancao |
| HBIN033150 | licoricesaponin j2 | Gancao |
| HBIN033151 | licorice-saponin J2 | Gancao |
| HBIN033152 | licorice-saponin J2_qt | Gancao |
| HBIN033153 | licoricesaponin k2 | Gancao |
| HBIN033154 | licorice-saponin K2 | Gancao |
| HBIN033155 | licorice-saponin K2_qt | Gancao |
| HBIN033156 | Licoricidin | Gancao |
| HBIN033157 | Licoricone | Gancao |
| HBIN033158 | Licoriisoflavan A | Gancao |
| HBIN033159 | licorisoflavan a | Gancao |
| HBIN033160 | licuraside | Gancao |
| HBIN033374 | liquiriligenin | Gancao |
| HBIN033376 | liquiritigenin | Gancao |
| HBIN033377 | liquiritigenin4'-o-β-d-apio-d-furanosyl(1→2)-β-d-glucopyranoside | Gancao |
| HBIN033378 | liquiritigenin-7,4'-diglucoside | Gancao |
| HBIN033379 | liquiritigenin-7-o-β-d-(3-o-acetyl)-apio-furanosyl-4'-o-β-d-glucopyranoside | Gancao |
| HBIN033383 | liquiritin | Gancao |
| HBIN033384 | Liquiritin apioside | Gancao |
| HBIN033386 | liquoric acid | Gancao |
| HBIN033768 | Lupiwighteone | Gancao |
| HBIN034623 | Medicarpin | Gancao |
| HBIN034629 | (-)-Medicocarpin | Gancao |
| HBIN034892 | methyl 18α-hydroxyglycyrrhetate | Gancao |
| HBIN034924 | methyl-24-hydroxy-11-deoxoglycyrrhetate | Gancao |
| HBIN034925 | methyl-24-hydroxyglycyrrhetate | Gancao |
| HBIN034960 | methyl 2-hydroxy-3,4-dimethoxybenzoate | Gancao |
| HBIN034997 | methyl 3-O-β-D-glucopyranosyl polygalacate | Gancao |
| HBIN035161 | Methylcyclopentane | Gancao |
| HBIN035245 | methylglycyrrhetate | Gancao |
| HBIN035246 | methylglyoxal | Gancao |
| HBIN035257 | Methylheptane | Gancao |
| HBIN035296 | methyl linoleate | Gancao |
| HBIN035318 | methyl-n-butyl-uralsaponin a esters | Gancao |
| HBIN035573 | Mipax | Gancao |
| HBIN035670 | monoammonium glycyrrhizinate | Gancao |
| HBIN035791 | morusin | Gancao |
| HBIN036347 | narcissin | Gancao |
| HBIN036349 | Narcissoside | Gancao |
| HBIN036366 | naringenin | Gancao |
| HBIN036380 | naringin | Gancao |
| HBIN036383 | narwedine | Gancao |
| HBIN036639 | neohancoside a | Gancao |
| HBIN036658 | neoisoliquiritin | Gancao |
| HBIN036661 | neoisopulegol | Gancao |
| HBIN036683 | neoliquiritin | Gancao |
| HBIN036689 | neomatatabiol | Gancao |
| HBIN036760 | Neouralenol | Gancao |
| HBIN036764 | neowilforine | Gancao |
| HBIN037389 | Nortangeretin | Gancao |
| HBIN037559 | n-tricosane | Gancao |
| HBIN037715 | OCT | Gancao |
| HBIN037777 | Octadiene | Gancao |
| HBIN037827 | odoratin | Gancao |
| HBIN038168 | ononin | Gancao |
| HBIN038169 | ononitol | Gancao |
| HBIN038485 | o-xylene | Gancao |
| HBIN039146 | Pentadecanol | Gancao |
| HBIN039185 | PENTYLFURAN | Gancao |
| HBIN039409 | Phaseol | Gancao |
| HBIN039411 | Phaseolinisoflavan | Gancao |
| HBIN039416 | phaseollinisoflavan | Gancao |
| HBIN039420 | phebalosin | Gancao |
| HBIN039998 | Pinocembrin | Gancao |
| HBIN040966 | prunetin | Gancao |
| HBIN041334 | p-xylene | Gancao |
| HBIN041495 | quercetin | Gancao |
| HBIN041709 | Quercetin der. | Gancao |
| HBIN042670 | rutin | Gancao |
| HBIN042674 | Ruvoside | Gancao |
| HBIN043316 | schaftoside | Gancao |
| HBIN043672 | Semilicoisoflavone B | Gancao |
| HBIN043855 | Sextone B | Gancao |
| HBIN043917 | shinpterocarpin | Gancao |
| HBIN044015 | sigmoidin b | Gancao |
| HBIN044016 | Sigmoidin-B | Gancao |
| HBIN044062 | sinapic acid | Gancao |
| HBIN044462 | soy lecithin | Gancao |
| HBIN046074 | tetrahydroharmine | Gancao |
| HBIN046084 | tetrahydropalmatine | Gancao |
| HBIN047564 | uralenin | Gancao |
| HBIN047565 | uralenneoside | Gancao |
| HBIN047566 | Uralenol | Gancao |
| HBIN047567 | Uralenol-3-methylether | Gancao |
| HBIN047569 | uralsaponin a | Gancao |
| HBIN047570 | uralsaponin B | Gancao |
| HBIN047571 | uralstilbene | Gancao |
| HBIN047573 | urea | Gancao |
| HBIN047613 | ursolic acid | Gancao |
| HBIN047875 | Vestitol | Gancao |
| HBIN047905 | Vicenin-2 | Gancao |
| HBIN047908 | vicianin | Gancao |
| HBIN047980 | violanthin | Gancao |
| HBIN048018 | viscotoxin a3 | Gancao |
| HBIN048051 | vitamin e | Gancao |
| HBIN048102 | vitexin | Gancao |
| HBIN048330 | WLN: 4OVR | Gancao |
| HBIN048417 | Xambioona | Gancao |
| HBIN048633 | Yinyanghuo D | Gancao |
| HBIN048716 | (Z)-1-(2,4-dihydroxyphenyl)-3-phenylprop-2-en-1-one | Gancao |
| HBIN000215 | 1, 10, 14-trimethyl-2-pentadecanone | Guizhi |
| HBIN000733 | 1,2-Benzenedicarboxylicacid, mono(2-ethyl) hexylester | Guizhi |
| HBIN000781 | 1,2-Dibenzoylethane | Guizhi |
| HBIN001393 | 1,4-cadinadiene | Guizhi |
| HBIN002159 | 19435-97-3 | Guizhi |
| HBIN003101 | (1R,3R,4S)-3,4-dimethylcyclohexan-1-ol | Guizhi |
| HBIN003102 | (1R,3R,5R)-6,6-dimethyl-2-methylene-3-norpinanol | Guizhi |
| HBIN003117 | (1R,4aR,8aS)-1-isopropyl-7-methyl-4-methylene-2,3,4a,5,6,8a-hexahydro-1H-naphthalene | Guizhi |
| HBIN003125 | (1R,4R)-4-isopropyl-1,6-dimethyltetralin | Guizhi |
| HBIN003153 | (1R,5R,7S)-4,7-dimethyl-7-(4-methylpent-3-enyl)bicyclo[3.1.1]hept-3-ene | Guizhi |
| HBIN003165 | (1R,8aS)-4-isopropyl-1,6-dimethyl-1,2,3,7,8,8a-hexahydronaphthalene | Guizhi |
| HBIN003276 | (1S,4R)-1,7,7-trimethylbicyclo[2.2.1]hept-2-ene | Guizhi |
| HBIN003278 | (1S,4R,4aR,8aR)-1-isopropyl-4,7-dimethyl-2,3,4,5,6,8a-hexahydro-1H-naphthalen-4a-ol | Guizhi |
| HBIN003284 | (1S,4R)-fenchone | Guizhi |
| HBIN003326 | [(1S)-endo]-(-)-Borneol | Guizhi |
| HBIN003849 | 2',3,4,4'-tetrahydrochalcone | Guizhi |
| HBIN003980 | (+-)-2,3-dihydro-2-(1-methylethenyl)-5-benzofurancarboxylic acid methyl ester | Guizhi |
| HBIN004822 | 2,6,10,15-tetramethylheptadecane | Guizhi |
| HBIN005474 | 2-Coumarate | Guizhi |
| HBIN005475 | 2-Coumarinate | Guizhi |
| HBIN005600 | 2-ethoxypropanol | Guizhi |
| HBIN005791 | 2'-hydroxycinnamaldehyde | Guizhi |
| HBIN005906 | 2-Methoxycin namaldehyde | Guizhi |
| HBIN005907 | 2-Methoxycinnamic Acid | Guizhi |
| HBIN005924 | 2-Methoxyphenylacetone | Guizhi |
| HBIN006077 | 2-Methyl-N-phenylmaleimide | Guizhi |
| HBIN006323 | 2-Phenylpropenal | Guizhi |
| HBIN007391 | 3,4-dihydroxybenzoicacid | Guizhi |
| HBIN007474 | 3-(4-hdroxyphenyl)-trans-propenoic acid-2,3-dihydroxypropyl ester | Guizhi |
| HBIN009375 | 3-o-p-hydroxy-trans-cinnamoylmaslinic acid | Guizhi |
| HBIN009514 | (3R,4aR,8aR)-3-isopropenyl-5,8a-dimethyl-2,3,4,4a,7,8-hexahydro-1H-naphthalene | Guizhi |
| HBIN010236 | (4aR,9aS)-2,9,9-trimethyl-5-methylene-4,4a,6,7,8,9a-hexahydro-3H-benzo[7]annulene | Guizhi |
| HBIN010661 | 4-Methyl-2-(1,5-dimethyl-4-hexenyl)-3-cyclohexen-1-ol | Guizhi |
| HBIN010881 | (4S)-4-[(1Z)-1,5-dimethylhexa-1,4-dienyl]-1-methylcyclohexene | Guizhi |
| HBIN011333 | 58870_FLUKA | Guizhi |
| HBIN011501 | 5-cinnamoyl-9-o-acetylphototaxicin i | Guizhi |
| HBIN012145 | 694-87-1 | Guizhi |
| HBIN013032 | 7alpha,21S,25-Trihydroxy-3beta-acetoxy-21S,23R-epoxy-9(11)-en-dammarane | Guizhi |
| HBIN013573 | (&#8722;)-Alloaromadendrene | Guizhi |
| HBIN015420 | alpha cadinene | Guizhi |
| HBIN015421 | α-Cadinene | Guizhi |
| HBIN015524 | ()-alpha-Funebrene | Guizhi |
| HBIN015588 | ()-alpha-Longipinene | Guizhi |
| HBIN015590 | α-Longipinene | Guizhi |
| HBIN015613 | α-muurolene | Guizhi |
| HBIN016156 | anhydrocinnzeylanine | Guizhi |
| HBIN016157 | anhydrocinnzeylanol | Guizhi |
| HBIN016180 | anisaldehyde | Guizhi |
| HBIN016527 | Apple oil | Guizhi |
| HBIN017729 | belta-terinene | Guizhi |
| HBIN017734 | benzaldehyde | Guizhi |
| HBIN017775 | Benzenepropanol | Guizhi |
| HBIN017842 | Benzyl acetate | Guizhi |
| HBIN017904 | bergamotene (Z,.alpha.,cis) | Guizhi |
| HBIN017978 | beta-asarone | Guizhi |
| HBIN018033 | beta-Cubebene | Guizhi |
| HBIN018190 | beta-Methoxystyrene | Guizhi |
| HBIN018325 | beta-sitoterol | Guizhi |
| HBIN018534 | Biosol | Guizhi |
| HBIN018743 | ()-Bornyl acetate | Guizhi |
| HBIN019199 | BZM | Guizhi |
| HBIN019239 | Cadalin | Guizhi |
| HBIN019321 | calacorene | Guizhi |
| HBIN019550 | cannabichromene | Guizhi |
| HBIN019551 | cannabichromenic acid | Guizhi |
| HBIN019558 | cannabidiol | Guizhi |
| HBIN019559 | cannabidiolic acid | Guizhi |
| HBIN019571 | Cannabinol | Guizhi |
| HBIN019572 | cannabinolic acid | Guizhi |
| HBIN019810 | caryophellene | Guizhi |
| HBIN019826 | (-)-Caryophyllene oxide | Guizhi |
| HBIN020162 | Cerulignol | Guizhi |
| HBIN020247 | chavicol | Guizhi |
| HBIN020653 | cinnamaldehyde | Guizhi |
| HBIN020657 | cinnamic acid | Guizhi |
| HBIN020663 | cinnamicalcohol | Guizhi |
| HBIN020664 | cinnamic aldehyde | Guizhi |
| HBIN020677 | cinnamyl acetate | Guizhi |
| HBIN020837 | cis-Cinnamaldehyde | Guizhi |
| HBIN020969 | cis-Zimtsaeure | Guizhi |
| HBIN021138 | Clorius | Guizhi |
| HBIN021608 | coumarin | Guizhi |
| HBIN021611 | coumarinic acid | Guizhi |
| HBIN022587 | dalbergin | Guizhi |
| HBIN023172 | delta2-tetrahydrocannabinolic acid | Guizhi |
| HBIN023188 | delta(9)-tetrahydrocannabinol | Guizhi |
| HBIN023404 | DEP | Guizhi |
| HBIN023925 | dihydromelilotoside | Guizhi |
| HBIN024334 | DMEP | Guizhi |
| HBIN024452 | d-Piperitone | Guizhi |
| HBIN024930 | EIC | Guizhi |
| HBIN024967 | ELD | Guizhi |
| HBIN025185 | ent-Epicatechin | Guizhi |
| HBIN025249 | epi-10- .gamma.-Eudesmol | Guizhi |
| HBIN025903 | Ethylcinnamate | Guizhi |
| HBIN025949 | Ethyl methoxycinnamate | Guizhi |
| HBIN026385 | farnesol | Guizhi |
| HBIN026486 | fetidine | Guizhi |
| HBIN027109 | .gamma.-Bisabolene | Guizhi |
| HBIN027528 | geraniol | Guizhi |
| HBIN028411 | Green Oil | Guizhi |
| HBIN028476 | Guaiol | Guizhi |
| HBIN028832 | HCI | Guizhi |
| HBIN029055 | Hepanal | Guizhi |
| HBIN029061 | HEPTACOSANE | Guizhi |
| HBIN029101 | heptanoic acid | Guizhi |
| HBIN029485 | Homocresol | Guizhi |
| HBIN029648 | Hyacinthin | Guizhi |
| HBIN029663 | Hydro Cinnamicacid | Guizhi |
| HBIN030414 | Isoamyl benzoate | Guizhi |
| HBIN030644 | isodalbergin | Guizhi |
| HBIN030806 | Isohomogenol | Guizhi |
| HBIN031387 | Iva | Guizhi |
| HBIN033803 | luteolin | Guizhi |
| HBIN034599 | m-Cymol | Guizhi |
| HBIN034685 | meliloticacid | Guizhi |
| HBIN034686 | melilotocarpan a | Guizhi |
| HBIN035142 | Methylcinnamate | Guizhi |
| HBIN035468 | Methyl (Z)-cinnamate | Guizhi |
| HBIN035481 | m-Formylphenol | Guizhi |
| HBIN035687 | mononetin | Guizhi |
| HBIN036009 | muscarine | Guizhi |
| HBIN036012 | muscarine ii | Guizhi |
| HBIN036015 | musclide a1 | Guizhi |
| HBIN036311 | Naphthalene, 1,2,3,4,4a,5,6,8a-octahydro-7-methyl-4-methylene-1-(1-methylethyl)-, (1alpha,4abeta,8aalpha)- | Guizhi |
| HBIN037053 | NK | Guizhi |
| HBIN037254 | nonanoic acid | Guizhi |
| HBIN037315 | nordalbergin | Guizhi |
| HBIN037630 | o-Acetyltoluene | Guizhi |
| HBIN037635 | o-Anisaldehyde | Guizhi |
| HBIN037636 | o-Anisic acid | Guizhi |
| HBIN037683 | obtustyrene | Guizhi |
| HBIN037738 | Octadecanal | Guizhi |
| HBIN037842 | odoriflavone | Guizhi |
| HBIN037902 | Oktadekan | Guizhi |
| HBIN038097 | O-METHOXYCINNAMALDEHYDE | Guizhi |
| HBIN038800 | Papite | Guizhi |
| HBIN039070 | PEL | Guizhi |
| HBIN039303 | Peroxyergosterol | Guizhi |
| HBIN039336 | Peruviol | Guizhi |
| HBIN039419 | PHB | Guizhi |
| HBIN039457 | Phenethyl acetate | Guizhi |
| HBIN039794 | phytol | Guizhi |
| HBIN040328 | p-Methoxycinnamaldehyde | Guizhi |
| HBIN040747 | proanthocyanidin b2 | Guizhi |
| HBIN040905 | protocatechualdehyde | Guizhi |
| HBIN040909 | Protocatechuic acid-3-glucoside | Guizhi |
| HBIN041428 | Pyruvophenone | Guizhi |
| HBIN041810 | (R)-2-methylbutyric acid | Guizhi |
| HBIN044684 | ST069309 | Guizhi |
| HBIN044783 | Stenol | Guizhi |
| HBIN045028 | styrene | Guizhi |
| HBIN045031 | Styrone | Guizhi |
| HBIN045055 | Substance H 36 | Guizhi |
| HBIN045118 | Sulcatone | Guizhi |
| HBIN045577 | tau-cadinol | Guizhi |
| HBIN045672 | taxifolin | Guizhi |
| HBIN045673 | (-)-taxifolin | Guizhi |
| HBIN045972 | ()-Terpinen-4-ol | Guizhi |
| HBIN046019 | Tetracosane | Guizhi |
| HBIN046031 | tetradecanal | Guizhi |
| HBIN046058 | tetrahydrocannabinol | Guizhi |
| HBIN046387 | thymol | Guizhi |
| HBIN046497 | T-Muurolol | Guizhi |
| HBIN046522 | Tolualdehydes | Guizhi |
| HBIN046738 | trans-cinnamic acid | Guizhi |
| HBIN047060 | trigonelline | Guizhi |
| HBIN048328 | WLN: 2OVR | Guizhi |
| HBIN048329 | WLN: 2VR | Guizhi |
| HBIN048346 | WLN: QR BV1 | Guizhi |
| HBIN048358 | WLN: RVO2R | Guizhi |
| HBIN048365 | WLN: VH2R | Guizhi |
| HBIN048367 | WLN: VHO2R | Guizhi |
| HBIN048368 | WLN: VHR | Guizhi |
| HBIN048717 | (Z)-1,3-di(phenyl)prop-2-en-1-one | Guizhi |
| HBIN048860 | zeatin | Guizhi |
| HBIN048888 | (Z)-Ethyl cinnamate | Guizhi |
| HBIN049092 | (Z,Z)-farnesol | Guizhi |
| HBIN049178 | Δ1-tetrahydrocannabinolicacid a | Guizhi |
| HBIN049200 | Δ9-tetrahydrocannabinol | Guizhi |
| HBIN037549 | n-trans-feruloyltyramine | Guizhi, Dazao |
| HBIN038026 | oleic acid | Guizhi, Dazao |
| HBIN038995 | p-coumaric acid | Guizhi, Dazao |
| HBIN015470 | α-cubebol | Guizhi, Gancao |
| HBIN016080 | anethole | Guizhi, Gancao |
| HBIN023623 | DIBP | Guizhi, Gancao |
| HBIN027159 | gamma-sitosterol | Guizhi, Gancao |
| HBIN034746 | ()-Menthol | Guizhi, Gancao |
| HBIN040908 | protocatechuic acid | Guizhi, Gancao |
| HBIN014399 | acetic acid | Guizhi, Shaoyao |
| HBIN015447 | (-)-alpha-cedrene | Guizhi, Shaoyao |
| HBIN018781 | BOX | Guizhi, Shaoyao |
| HBIN019921 | (+)-catechin | Guizhi, Shaoyao, Dazao |
| HBIN022815 | DBP | Guizhi, Shaoyao, Gancao |
| HBIN019502 | camphor | Guizhi, Shaoyao, Xixin |
| HBIN036803 | nerolidol | Guizhi, Shaoyao, Xixin |
| HBIN044730 | stearic acid | Guizhi, Shaoyao, Xixin, Dazao |
| HBIN044158 | sitosterol | Guizhi, Tongcao, Gancao |
| HBIN002102 | 1,8 cineole | Guizhi, Xixin |
| HBIN002161 | 19894-97-4 | Guizhi, Xixin |
| HBIN002561 | 1H-Cycloprop(e)azulen-7-ol, decahydro-1,1,7-trimethyl-4-methylene-, (1aR-(1aalpha,4aalpha,7beta,7abeta,7balpha))- | Guizhi, Xixin |
| HBIN003134 | (1R,4S,4aR,8aR)-4-isopropyl-1,6-dimethyl-3,4,4a,7,8,8a-hexahydro-2H-naphthalen-1-ol | Guizhi, Xixin |
| HBIN003295 | (1S,4S)-7-isopropylidene-1,4-dimethyl-2,3,4,5,6,8-hexahydro-1H-azulene | Guizhi, Xixin |
| HBIN003373 | (1Z,4E,8E)-2,6,6,9-tetramethylcycloundeca-1,4,8-triene | Guizhi, Xixin |
| HBIN011920 | (5S)-1-isopropyl-4-methylbicyclo[3.1.0]hex-3-ene | Guizhi, Xixin |
| HBIN015469 | alpha-Cubebene | Guizhi, Xixin |
| HBIN015476 | alpha-curcumene | Guizhi, Xixin |
| HBIN015700 | alpha-terpinene | Guizhi, Xixin |
| HBIN016887 | ()-Aromadendrene | Guizhi, Xixin |
| HBIN018231 | (-)-beta-Phellandrene | Guizhi, Xixin |
| HBIN018241 | ()-beta-Pinene | Guizhi, Xixin |
| HBIN018729 | (-)-Borneol | Guizhi, Xixin |
| HBIN019449 | CAM | Guizhi, Xixin |
| HBIN019688 | caprylic acid | Guizhi, Xixin |
| HBIN021312 | (-)-Comphene | Guizhi, Xixin |
| HBIN021422 | copaene | Guizhi, Xixin |
| HBIN023190 | delta-amorphene | Guizhi, Xixin |
| HBIN025445 | (-)-Epoxycaryophyllene | Guizhi, Xixin |
| HBIN026067 | eugenol | Guizhi, Xixin |
| HBIN029260 | hexadecane | Guizhi, Xixin |
| HBIN029313 | hexanal | Guizhi, Xixin |
| HBIN030497 | (+/-)-Isoborneol | Guizhi, Xixin |
| HBIN030562 | Isocaryophyllene | Guizhi, Xixin |
| HBIN032807 | L-Bornyl acetate | Guizhi, Xixin |
| HBIN033428 | L-Limonen | Guizhi, Xixin |
| HBIN036044 | muurolene | Guizhi, Xixin |
| HBIN036197 | MYS | Guizhi, Xixin |
| HBIN036819 | neryl acetate | Guizhi, Xixin |
| HBIN037281 | (-)-nopinene | Guizhi, Xixin |
| HBIN041914 | (R)-(-)-alpha-Phellandrene | Guizhi, Xixin |
| HBIN042339 | (R)-linalool | Guizhi, Xixin |
| HBIN042501 | (R)-p-Menth-1-en-4-ol | Guizhi, Xixin |
| HBIN045254 | Syringaldehyde | Guizhi, Xixin |
| HBIN045966 | Terpilene | Guizhi, Xixin |
| HBIN045982 | Terragon | Guizhi, Xixin |
| HBIN048841 | (Z)-calamenene | Guizhi, Xixin |
| HBIN048935 | ZINC01609418 | Guizhi, Xixin |
| HBIN049119 | γ-elemene | Guizhi, Xixin |
| HBIN022819 | d-camphene | Guizhi, Xixin, Dazao |
| HBIN035214 | methyleugenol | Guizhi, Xixin, Dazao |
| HBIN032583 | (L)-alpha-Terpineol | Guizhi, Xixin, Gancao |
| HBIN000170 | 10-Methylnonadecane | Shaoyao |
| HBIN000205 | 10-undecenoicacid | Shaoyao |
| HBIN000337 | 11alpha,12alpha-epoxy-3beta-23-dihydroxy-30-norolean-20-en-28,12beta-olide | Shaoyao |
| HBIN000409 | 1,1-Dimethyl-2-(3-methyl-1,3-butadiene)-cyclo-propane | Shaoyao |
| HBIN000573 | 1,2,3,4,6-pentagalloylglucose | Shaoyao |
| HBIN000606 | 1,2,3,6-tetra-O-galloyl-β-D-glucose | Shaoyao |
| HBIN000607 | 1,2,3,6-tetra-O-galloylglucose | Shaoyao |
| HBIN000626 | 1,2,3-Tri-O-galloyl-beta-D-glucose | Shaoyao |
| HBIN000689 | 1,2,6-tri-O-galloyl-β-D-glucose | Shaoyao |
| HBIN000912 | 12-o-2z,4e-octadienoyl-4-dexyphorbol-13-acetate | Shaoyao |
| HBIN001086 | 1,3,6-trigalloyl-β-d-glucose | Shaoyao |
| HBIN001263 | 13-Methyl pentadecanoic acid | Shaoyao |
| HBIN001265 | 13-methyl tetradecanoicacid | Shaoyao |
| HBIN001343 | 1,4,7-tridecatriene | Shaoyao |
| HBIN002568 | 1-heptadecanol | Shaoyao |
| HBIN002758 | 1-methyl-2-[(z)-6-pentadecenyl]-4(1h)-quinolone | Shaoyao |
| HBIN002818 | 1-monolinolein | Shaoyao |
| HBIN002834 | 1-nonen-3-ol | Shaoyao |
| HBIN002910 | 1-o-galloyl-glucose | Shaoyao |
| HBIN002955 | 1-pentadecene | Shaoyao |
| HBIN003176 | (1R)-()-Nopinone | Shaoyao |
| HBIN003340 | 1-Tetradecene | Shaoyao |
| HBIN003709 | 2,2-dimethylcyclohexanol | Shaoyao |
| HBIN003948 | 2,3-butanediol | Shaoyao |
| HBIN004147 | 2,3-o-(s)-hexahydroxydiphenoyl-d-gluco-pyranose | Shaoyao |
| HBIN004191 | 24253-30-3 | Shaoyao |
| HBIN004459 | 24-Methylenecycloartanol | Shaoyao |
| HBIN005687 | 2-Hexyl-1-decanol | Shaoyao |
| HBIN005963 | 2 - methyl - 3 - (2 - propenyl) - phenol | Shaoyao |
| HBIN007288 | 3,4,5-trihydroxybenzoic acid | Shaoyao |
| HBIN007986 | (3aR,6S,7aR)-6-hydroxy-6-methyl-3-methylene-3a,4,7,7a-tetrahydrobenzofuran-2,5-dione | Shaoyao |
| HBIN008077 | 3β,23-dihydroxy-oleana-11,13(18)-dien-28-oic acid | Shaoyao |
| HBIN008218 | 3β-hydroxy-11-oxo-olean-12-en-28-oic acid | Shaoyao |
| HBIN008255 | 3β-hydroxy-oleana-11,13(18)-dien-28-oic acid? | Shaoyao |
| HBIN009505 | (3R,3aR,6S,7aR)-6-hydroxy-3,6-dimethyl-3a,4,7,7a-tetrahydro-3H-benzofuran-2,5-dione | Shaoyao |
| HBIN009602 | (3S,3aR,5S,6S,7aR)-5,6-dihydroxy-3,6-dimethyl-3,3a,4,5,7,7a-hexahydrobenzofuran-2-one | Shaoyao |
| HBIN009603 | [(3S,3aR,6S,7aR)-6-hydroxy-6-methyl-2,5-dioxo-3a,4,7,7a-tetrahydro-3H-benzofuran-3-yl]methyl benzoate | Shaoyao |
| HBIN009663 | (3S,5R,8R,9R,10S,14S)-3,17-dihydroxy-4,4,8,10,14-pentamethyl-2,3,5,6,7,9-hexahydro-1H-cyclopenta[a]phenanthrene-15,16-dione | Shaoyao |
| HBIN010296 | 4-Chlorobutyric acid | Shaoyao |
| HBIN010770 | 4-o-galloyl quinicacid | Shaoyao |
| HBIN011516 | 5-desgalloylstachyurin | Shaoyao |
| HBIN011554 | 5-(heptadec-12-enyl) resorcinol | Shaoyao |
| HBIN011858 | 5-O-Galloylquinic acid | Shaoyao |
| HBIN012763 | (6R,10R)-6,10,14-trimethylpentadecan-2-one | Shaoyao |
| HBIN013157 | 7-Demethylsuberosin | Shaoyao |
| HBIN013837 | 8-NONENOIC ACID | Shaoyao |
| HBIN014139 | 9-methylenefluorene | Shaoyao |
| HBIN014204 | 9-(z)-octadecen-1-ol | Shaoyao |
| HBIN014418 | acetoin | Shaoyao |
| HBIN014487 | Acetyl oxide | Shaoyao |
| HBIN015074 | albiflorin | Shaoyao |
| HBIN015078 | albiflorin_qt | Shaoyao |
| HBIN015079 | albiflorin R1 | Shaoyao |
| HBIN015080 | albiflorin R1_qt | Shaoyao |
| HBIN015224 | alloimperatorin | Shaoyao |
| HBIN015225 | alloimperatorine | Shaoyao |
| HBIN015226 | Alloisoimperatorin | Shaoyao |
| HBIN015534 | α-guriunene | Shaoyao |
| HBIN015536 | alpha-Guttiferin | Shaoyao |
| HBIN015547 | alpha-humulene | Shaoyao |
| HBIN017410 | aviprin | Shaoyao |
| HBIN017786 | benzoic acid | Shaoyao |
| HBIN017827 | benzoyl paeoniflorin | Shaoyao |
| HBIN018401 | Bicetyl | Shaoyao |
| HBIN018434 | bicyclo[3.1.1]hept-2-ene-2-methanol, 6,6-dimethyl- | Shaoyao |
| HBIN018742 | bornyl acetate | Shaoyao |
| HBIN018951 | BU3 | Shaoyao |
| HBIN019189 | Byakangelicin | Shaoyao |
| HBIN019195 | bya-kangelicol | Shaoyao |
| HBIN019903 | Casuarictin | Shaoyao |
| HBIN019904 | casuariin | Shaoyao |
| HBIN019918 | catechin | Shaoyao |
| HBIN020786 | cis-4-tetradecenoic acid | Shaoyao |
| HBIN020790 | cis-5-Octen-1-ol | Shaoyao |
| HBIN022567 | dahuribirin a | Shaoyao |
| HBIN022568 | dahuribirin b | Shaoyao |
| HBIN022569 | dahuribirin c | Shaoyao |
| HBIN022570 | dahuribirin d | Shaoyao |
| HBIN022571 | dahuribirin e | Shaoyao |
| HBIN022572 | dahuribirin f | Shaoyao |
| HBIN022573 | dahuribirin g | Shaoyao |
| HBIN022822 | d-catechin | Shaoyao |
| HBIN023261 | demethylteuicausine | Shaoyao |
| HBIN023630 | Dibutylphenol | Shaoyao |
| HBIN024228 | Dipropyl phthalate | Shaoyao |
| HBIN024367 | dodecanal | Shaoyao |
| HBIN024875 | EEE | Shaoyao |
| HBIN024973 | elemene | Shaoyao |
| HBIN025344 | epigallocatechin | Shaoyao |
| HBIN025872 | ethyl acetate | Shaoyao |
| HBIN025934 | Ethylisobutyrate | Shaoyao |
| HBIN025940 | Ethyllaurate | Shaoyao |
| HBIN025941 | ethylleptol b | Shaoyao |
| HBIN026064 | eugeniin | Shaoyao |
| HBIN026276 | Exaltolide | Shaoyao |
| HBIN026306 | exoticin | Shaoyao |
| HBIN027030 | gallic acid | Shaoyao |
| HBIN027045 | gallocatechin | Shaoyao |
| HBIN027057 | gallotannin | Shaoyao |
| HBIN027064 | galloylpaeoniflorin | Shaoyao |
| HBIN027547 | geranyl propionate | Shaoyao |
| HBIN028902 | Hederagenol | Shaoyao |
| HBIN029037 | Henicosane | Shaoyao |
| HBIN030053 | imperatorin | Shaoyao |
| HBIN030055 | imperatorine | Shaoyao |
| HBIN032533 | lactiflorin | Shaoyao |
| HBIN032755 | lauric acid,ethyl ester | Shaoyao |
| HBIN033041 | LFA | Shaoyao |
| HBIN034504 | marmesin | Shaoyao |
| HBIN034507 | Marmesine | Shaoyao |
| HBIN034508 | marmesinin | Shaoyao |
| HBIN035157 | methyl cyclodecane | Shaoyao |
| HBIN035159 | methylcyclohexane | Shaoyao |
| HBIN035198 | Methylenetanshinquinone | Shaoyao |
| HBIN035224 | Methylgallate | Shaoyao |
| HBIN035255 | methyl heptadecanoate | Shaoyao |
| HBIN035297 | Methyl linolelaidate | Shaoyao |
| HBIN035329 | methyl nonadecanoate | Shaoyao |
| HBIN035336 | methyl octadecadienoate | Shaoyao |
| HBIN035341 | methyl oenanthate | Shaoyao |
| HBIN035353 | methyl palmitate | Shaoyao |
| HBIN035435 | methyl tetradecanoate | Shaoyao |
| HBIN035683 | monomethyl-cis-hinokiresinol | Shaoyao |
| HBIN036568 | neobyakangelico l | Shaoyao |
| HBIN036574 | neocapillene | Shaoyao |
| HBIN037086 | n-methylephedrine | Shaoyao |
| HBIN037160 | n-nonane | Shaoyao |
| HBIN037195 | nodakenin | Shaoyao |
| HBIN037196 | nodakenitin | Shaoyao |
| HBIN037268 | nonyl cyclopropane | Shaoyao |
| HBIN037270 | nonyl ethyl ether | Shaoyao |
| HBIN037591 | n-undecyl acetate | Shaoyao |
| HBIN037721 | Octacosane | Shaoyao |
| HBIN037729 | octadec-9-ene | Shaoyao |
| HBIN037759 | octadecanol | Shaoyao |
| HBIN037760 | octadecanyl-3-methoxy-4-hydorxy benzeneacrylate | Shaoyao |
| HBIN037796 | octanol | Shaoyao |
| HBIN038492 | oxypaeoniflorin | Shaoyao |
| HBIN038494 | oxypeucedanin | Shaoyao |
| HBIN038496 | (+)-oxypeucedanin | Shaoyao |
| HBIN038498 | oxypeucedanin hydrate | Shaoyao |
| HBIN038501 | oxypeucedanin methanolate | Shaoyao |
| HBIN038502 | oxyphyllacinol | Shaoyao |
| HBIN038543 | pabulenol | Shaoyao |
| HBIN038547 | pachycarin a | Shaoyao |
| HBIN038596 | paeonianiin e | Shaoyao |
| HBIN038597 | paeonianin a | Shaoyao |
| HBIN038598 | paeonianin b | Shaoyao |
| HBIN038599 | paeonianin c | Shaoyao |
| HBIN038600 | paeonianin d | Shaoyao |
| HBIN038604 | paeoniflorgenone | Shaoyao |
| HBIN038605 | Paeoniflorigenone | Shaoyao |
| HBIN038606 | paeoniflorin | Shaoyao |
| HBIN038607 | paeoniflorin_qt | Shaoyao |
| HBIN038610 | paeonilactone a | Shaoyao |
| HBIN038612 | paeonilactone b | Shaoyao |
| HBIN038613 | paeonilactone c | Shaoyao |
| HBIN038614 | paeonin | Shaoyao |
| HBIN038627 | paeonol | Shaoyao |
| HBIN038629 | paeonoside | Shaoyao |
| HBIN038639 | palbinone | Shaoyao |
| HBIN039003 | p-cymen-2-ol | Shaoyao |
| HBIN039046 | pedunculagin | Shaoyao |
| HBIN039151 | Pentagalloylglucose | Shaoyao |
| HBIN039198 | Peonin | Shaoyao |
| HBIN039199 | peonol | Shaoyao |
| HBIN039451 | Phellopterin | Shaoyao |
| HBIN039458 | phenethyl alcohol | Shaoyao |
| HBIN040142 | Pisol | Shaoyao |
| HBIN040797 | Progallin A | Shaoyao |
| HBIN040850 | propyl (2R)-2-hydroxypropanoate | Shaoyao |
| HBIN041268 | Pulchinenoside A_qt | Shaoyao |
| HBIN041339 | PYG | Shaoyao |
| HBIN041378 | pyrethrin i | Shaoyao |
| HBIN041379 | Pyrethrin II | Shaoyao |
| HBIN042852 | salicylic acid | Shaoyao |
| HBIN043212 | Satol | Shaoyao |
| HBIN043688 | sen-byakangelicol | Shaoyao |
| HBIN043692 | sendanone acetate | Shaoyao |
| HBIN044976 | strictinin | Shaoyao |
| HBIN045047 | suberosin | Shaoyao |
| HBIN045845 | tellimagrandin I | Shaoyao |
| HBIN045975 | terpinene-4-ol | Shaoyao |
| HBIN046783 | ()-trans-Myrtanol | Shaoyao |
| HBIN046867 | TRD | Shaoyao |
| HBIN047010 | tridecanoicacid | Shaoyao |
| HBIN047494 | umbelliprenin | Shaoyao |
| HBIN047539 | undecene | Shaoyao |
| HBIN048722 | (Z)-(1S,5R)-beta-pinen-10-yl-beta-vicianoside | Shaoyao |
| HBIN048724 | (Z)-(1S,5R)-beta-pinen-10-yl-beta-vicianoside_qt | Shaoyao |
| HBIN048725 | (z)-(1s,5r)-beta-pinen-1-oxy-beta-vicianoside | Shaoyao |
| HBIN048958 | ZINC02169908 | Shaoyao |
| HBIN019919 | (-)-catechin | Shaoyao, Dazao |
| HBIN045073 | sucrose | Shaoyao, Dazao |
| HBIN047492 | umbelliferone | Shaoyao, Gancao |
| HBIN034281 | Mairin | Shaoyao, Gancao, Dazao |
| HBIN037940 | oleanolic acid | Shaoyao, Gancao, Dazao |
| HBIN015117 | alexandrin | Shaoyao, Tongcao |
| HBIN015645 | alpha-phellandrene | Shaoyao, Xixin |
| HBIN029090 | Heptadekan | Shaoyao, Xixin |
| HBIN029604 | humulene | Shaoyao, Xixin |
| HBIN032812 | l-carvone | Shaoyao, Xixin |
| HBIN039006 | p-cymene | Shaoyao, Xixin |
| HBIN039150 | PENTADECYLIC ACID | Shaoyao, Xixin |
| HBIN043309 | (s)-carvone | Shaoyao, Xixin |
| HBIN045973 | terpinen-4-ol | Shaoyao, Xixin |
| HBIN046719 | trans-beta-farnesene | Shaoyao, Xixin |
| HBIN046736 | trans-caryophyllene | Shaoyao, Xixin |
| HBIN018242 | beta-pinene | Shaoyao, Xixin, Dazao |
| HBIN017212 | astragalin | Shaoyao, Xixin, Gancao |
| HBIN031753 | kaempferol | Shaoyao, Xixin, Gancao |
| HBIN015101 | aldehydo-D-galactose | Tongcao |
| HBIN016578 | Aralia cerebroside | Tongcao |
| HBIN016579 | Aralia cerebroside_qt | Tongcao |
| HBIN020163 | ceryl alcohol | Tongcao |
| HBIN027931 | gledinin | Tongcao |
| HBIN027932 | gleditschia saponin | Tongcao |
| HBIN030188 | inositol | Tongcao |
| HBIN030348 | iron | Tongcao |
| HBIN037220 | nonacosane | Tongcao |
| HBIN038812 | papyrioside L-Ⅱb | Tongcao |
| HBIN038813 | papyrioside L-Ⅱb_qt | Tongcao |
| HBIN038814 | papyrioside L-Ⅱc | Tongcao |
| HBIN038815 | papyrioside L-Ⅱc_qt | Tongcao |
| HBIN038816 | papyrioside L-Ⅱd | Tongcao |
| HBIN038817 | papyrioside L-Ⅱd_qt | Tongcao |
| HBIN038818 | papyrioside L- IIa | Tongcao |
| HBIN038819 | papyrioside L- IIa_qt | Tongcao |
| HBIN038897 | paryriogenin A | Tongcao |
| HBIN038898 | paryriogenin B | Tongcao |
| HBIN038899 | paryriogenin C | Tongcao |
| HBIN038900 | paryriogenin D | Tongcao |
| HBIN038901 | paryriogenin E | Tongcao |
| HBIN038902 | paryriogenin G | Tongcao |
| HBIN038903 | paryriogenin H | Tongcao |
| HBIN038904 | paryriogenin I | Tongcao |
| HBIN038905 | paryriogenin J | Tongcao |
| HBIN040830 | propapyriogeninA1 | Tongcao |
| HBIN040831 | propapyriogeninA2 | Tongcao |
| HBIN045487 | tannins | Tongcao |
| HBIN046114 | Tetrapanamide A | Tongcao |
| HBIN046115 | Tetrapanoside A | Tongcao |
| HBIN046116 | Tetrapanoside A_qt | Tongcao |
| HBIN046117 | Tetrapanoside B | Tongcao |
| HBIN046118 | Tetrapanoside B_qt | Tongcao |
| HBIN048512 | XLS | Tongcao |
| HBIN027963 | GLO | Tongcao, Dazao |
| HBIN036221 | N-(2'-hydroxytetraeosanol)-2-amino-8-octadecene-1,3,4-triol | Tongcao, Dazao |
| HBIN000620 | 1,2,3-trimethoxy-5-methyl benzene | Xixin |
| HBIN001034 | 1,3,4-trimethyl-3-cyclohexene-1-carboxaldehyde | Xixin |
| HBIN002179 | 1,9-Decadiyne | Xixin |
| HBIN002331 | 1-Asarinine | Xixin |
| HBIN002584 | 1-hexanol | Xixin |
| HBIN002956 | 1-Pentadecyne | Xixin |
| HBIN003022 | (1R)-1-phenylpropan-1-ol | Xixin |
| HBIN003109 | (1R,3S,5R)-6,6-dimethyl-2-methylene-3-norpinanol | Xixin |
| HBIN003246 | [(1S)-3-[(E)-but-2-enyl]-2-methyl-4-oxo-1-cyclopent-2-enyl] (1R,3R)-3-[(E)-3-methoxy-2-methyl-3-oxoprop-1-enyl]-2,2-dimethylcyclopropane-1-carboxylate | Xixin |
| HBIN003249 | (1S,3R,5S)-6,6-dimethyl-2-methylene-3-norpinanol | Xixin |
| HBIN003281 | (1S,4R,5R)-1-isopropyl-4-methyl-4-bicyclo[3.1.0]hexanol | Xixin |
| HBIN003303 | (1S,5R)-7,7-dimethyl-4-bicyclo[3.1.1]hept-3-enecarboxaldehyde | Xixin |
| HBIN003305 | (1S,5S)-1-isopropyl-4-methylenebicyclo[3.1.0]hexane | Xixin |
| HBIN003597 | 2-[(1R)-2,2,3-trimethyl-1-cyclopent-3-enyl]ethanal | Xixin |
| HBIN003599 | 2-[(1R,3S,4S)-3-isopropenyl-4-methyl-4-vinylcyclohexyl]propan-2-ol | Xixin |
| HBIN003602 | 2-[(1R)-4-methyl-1-cyclohex-3-enyl]propan-2-yl acetate | Xixin |
| HBIN003923 | 2,3,5-trimethoxytoluene | Xixin |
| HBIN003956 | 2,3-dehydro-1,8-cineole | Xixin |
| HBIN004358 | 2,4-Dimethylhexane | Xixin |
| HBIN005857 | 2-isopropyl-5-methylanisole | Xixin |
| HBIN005958 | 2-methyl-2-vinyl-3-isopropenyl-5-isopropylidene cyclohexanol | Xixin |
| HBIN007305 | 3,4,5-Trimethoxytoluene | Xixin |
| HBIN007658 | 3,5-Dimethoxytoluene | Xixin |
| HBIN007703 | 3691-11-0 | Xixin |
| HBIN008365 | 3-Butynol | Xixin |
| HBIN008894 | 3-Methyl-6-(1-methylethyl)-cyclohexene | Xixin |
| HBIN009370 | 3'-o-methylviolanone | Xixin |
| HBIN009695 | (3S)-7-hydroxy-3-(2,3,4-trimethoxyphenyl)chroman-4-one | Xixin |
| HBIN010154 | 4,9-dimethoxy-1-vinyl-$b-carboline | Xixin |
| HBIN010155 | 4,9-dimethoxy-1-vinyl-beta-carboline | Xixin |
| HBIN010235 | (4aR,8aS)-7-isopropylidene-4a-methyl-1-methylenedecalin | Xixin |
| HBIN010620 | 4-methoxy-6-[(E)-prop-1-enyl]-1,3-benzodioxole | Xixin |
| HBIN010863 | [(4R)-4-isopropenyl-1-cyclohexenyl]methanol | Xixin |
| HBIN011334 | 58924_FLUKA | Xixin |
| HBIN011726 | 5-isopropyl-2-methylbicyclo[3.1.0]hex-2-ene | Xixin |
| HBIN012103 | 6892-80-4 | Xixin |
| HBIN013422 | 7-oxabicyclo-2.2.1-heptane,1-methyl-4-[1-methylethyl]- | Xixin |
| HBIN014406 | ACETIC ACID,BORNYL ESTER | Xixin |
| HBIN014458 | acetylborneol | Xixin |
| HBIN014568 | ACon1_002321 | Xixin |
| HBIN015386 | alpha-asarone | Xixin |
| HBIN015410 | alpha-bisabolol | Xixin |
| HBIN015515 | alpha-Farnesene | Xixin |
| HBIN015533 | alpha-Guaiene | Xixin |
| HBIN015580 | alpha-limonene | Xixin |
| HBIN015710 | alpha-terpinolene | Xixin |
| HBIN016789 | aristolene | Xixin |
| HBIN016798 | aristolochicacid | Xixin |
| HBIN016955 | Artemisia triene | Xixin |
| HBIN017024 | asaricin | Xixin |
| HBIN017025 | asarinin | Xixin |
| HBIN017440 | Azaron | Xixin |
| HBIN017652 | BB_NC-0668 | Xixin |
| HBIN017992 | beta-Bourbonene | Xixin |
| HBIN018026 | beta-Citronellol | Xixin |
| HBIN018118 | (-)-beta-Fenchol | Xixin |
| HBIN018138 | beta-Gurjunene | Xixin |
| HBIN018261 | β-seisquiphellandrene | Xixin |
| HBIN018338 | β-terpineol | Xixin |
| HBIN018727 | borneol | Xixin |
| HBIN019112 | Butylbenzene | Xixin |
| HBIN019336 | calarene | Xixin |
| HBIN019699 | Car-3-ene | Xixin |
| HBIN019701 | (±)-car-3-ene-2,5-dione | Xixin |
| HBIN019740 | Caribine | Xixin |
| HBIN019791 | Carvacryl acetate | Xixin |
| HBIN020650 | cinerins | Xixin |
| HBIN020819 | cis-belta-terpineol | Xixin |
| HBIN020826 | cis-beta-farnesene | Xixin |
| HBIN020827 | cis-beta-Ocimene | Xixin |
| HBIN020999 | citronellyl formate | Xixin |
| HBIN021762 | Croweacin | Xixin |
| HBIN021764 | crpytopine | Xixin |
| HBIN021791 | Cryptopin | Xixin |
| HBIN022199 | CYCLODODECANE | Xixin |
| HBIN022249 | cyclohexene | Xixin |
| HBIN022266 | Cyclohexylmethanol | Xixin |
| HBIN022333 | Cyclopentadecane | Xixin |
| HBIN022445 | Cynanuriculoside A | Xixin |
| HBIN022446 | Cynanuriculoside A_qt | Xixin |
| HBIN022527 | d1-demethyl coclaurin | Xixin |
| HBIN022885 | dec-2-enal | Xixin |
| HBIN023103 | Dekan | Xixin |
| HBIN023204 | delta-elemene | Xixin |
| HBIN023213 | delta-Terpineol | Xixin |
| HBIN023537 | DHNQ | Xixin |
| HBIN024304 | dl-demethylcoclaurine | Xixin |
| HBIN024310 | d-limonene | Xixin |
| HBIN024321 | dl-Thujone | Xixin |
| HBIN024380 | Dodecatetraenamide, N-(2-methylpropyl)- | Xixin |
| HBIN024742 | (E)-9-Isopropyl-6-methyl-5,9-decadiene-2-one | Xixin |
| HBIN024933 | (E)-icos-9-ene | Xixin |
| HBIN024978 | Elemol | Xixin |
| HBIN025284 | epicamphor | Xixin |
| HBIN025406 | (+) epipinoresinol | Xixin |
| HBIN025818 | estragole | Xixin |
| HBIN025820 | Estriol | Xixin |
| HBIN026071 | eugenol methyl ether | Xixin |
| HBIN026072 | eugenone | Xixin |
| HBIN027107 | gamma-Asarone | Xixin |
| HBIN027161 | gamma-terpinene | Xixin |
| HBIN027559 | germacra-1(10),4,7(11)-trien-9α-ol | Xixin |
| HBIN027567 | Germacrene B | Xixin |
| HBIN027568 | Germacrene D | Xixin |
| HBIN029382 | higenamine | Xixin |
| HBIN029444 | HMF | Xixin |
| HBIN032004 | Kakoul | Xixin |
| HBIN032005 | kakuol | Xixin |
| HBIN033265 | linalool | Xixin |
| HBIN033269 | linalool oxide B (cis-THF) | Xixin |
| HBIN033279 | linalyl isobutyrate | Xixin |
| HBIN033440 | l-Menthone | Xixin |
| HBIN033895 | l-Verbenone | Xixin |
| HBIN034745 | menthol | Xixin |
| HBIN034853 | Methoxyeugenol | Xixin |
| HBIN034963 | (-)-Methyl 2-methylbutyrate | Xixin |
| HBIN035121 | Methyl butenone | Xixin |
| HBIN035415 | methyl salicylate | Xixin |
| HBIN036076 | Myricadiol | Xixin |
| HBIN036156 | Myristelaidic acid | Xixin |
| HBIN036167 | myristoleicacid | Xixin |
| HBIN036323 | naphthazarin | Xixin |
| HBIN036445 | n-butyl-β-D-fructopyronoside | Xixin |
| HBIN036795 | nerol | Xixin |
| HBIN036894 | n-ibdta | Xixin |
| HBIN037037 | n-isobutyldodecatetramine | Xixin |
| HBIN037485 | n-pentadecane | Xixin |
| HBIN037763 | OCTADECENE | Xixin |
| HBIN037814 | Octyl formate | Xixin |
| HBIN038132 | O-Methylthymol | Xixin |
| HBIN038534 | OYA | Xixin |
| HBIN038919 | patchouli alcohol | Xixin |
| HBIN039004 | p-Cymen-8-ol | Xixin |
| HBIN039143 | pentadecanoic acid | Xixin |
| HBIN039147 | Pentadecene | Xixin |
| HBIN039995 | pinocarvone | Xixin |
| HBIN040075 | piperitol | Xixin |
| HBIN041273 | pulegone | Xixin |
| HBIN041285 | pulvratilol | Xixin |
| HBIN042691 | (S)-2,2,3-Trimethylcyclopent-3-ene-1-acetaldehyde | Xixin |
| HBIN042726 | sabinene | Xixin |
| HBIN042727 | sabinene hydrate | Xixin |
| HBIN042762 | Safynol | Xixin |
| HBIN043162 | Sarisan | Xixin |
| HBIN043173 | Sarmentoloside | Xixin |
| HBIN043798 | sesamin | Xixin |
| HBIN043802 | sesamol | Xixin |
| HBIN045937 | Tereben | Xixin |
| HBIN045980 | terpinolene | Xixin |
| HBIN045981 | terpinyl acetate | Xixin |
| HBIN046479 | Tip-Nip | Xixin |
| HBIN046495 | TML | Xixin |
| HBIN046731 | (-)-trans-Carveol | Xixin |
| HBIN046798 | trans-p-2-Menthen-1-ol | Xixin |
| HBIN046860 | trans-Verbenol | Xixin |
| HBIN047124 | trimethylamine | Xixin |
| HBIN048952 | ZINC02040970 | Xixin |
| HBIN048956 | ZINC02140511 | Xixin |
| HBIN048981 | ZINC05223929 | Xixin |
| HBIN049077 | Zyklohexen | Xixin |
| HBIN049221 | δ-selinene | Xixin |
| HBIN024976 | elemicin | Xixin, Dazao |
| HBIN042761 | safrole | Xixin, Dazao |
| HBIN036050 | m-xylene | Xixin, Gancao |
| HBIN036903 | nicotiflorin | Xixin, Gancao |

Supplemental Figure 1


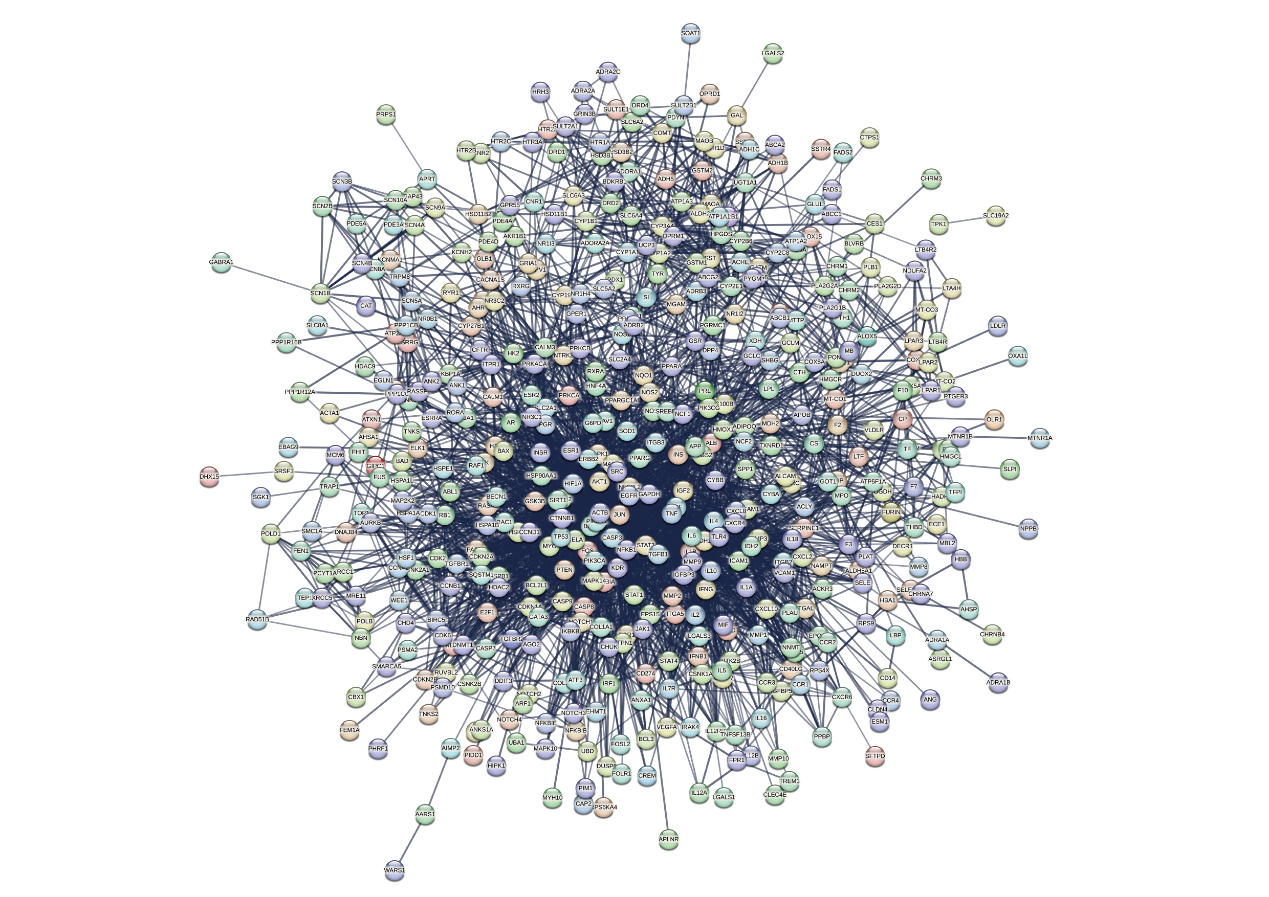


Supplemental Figure 2


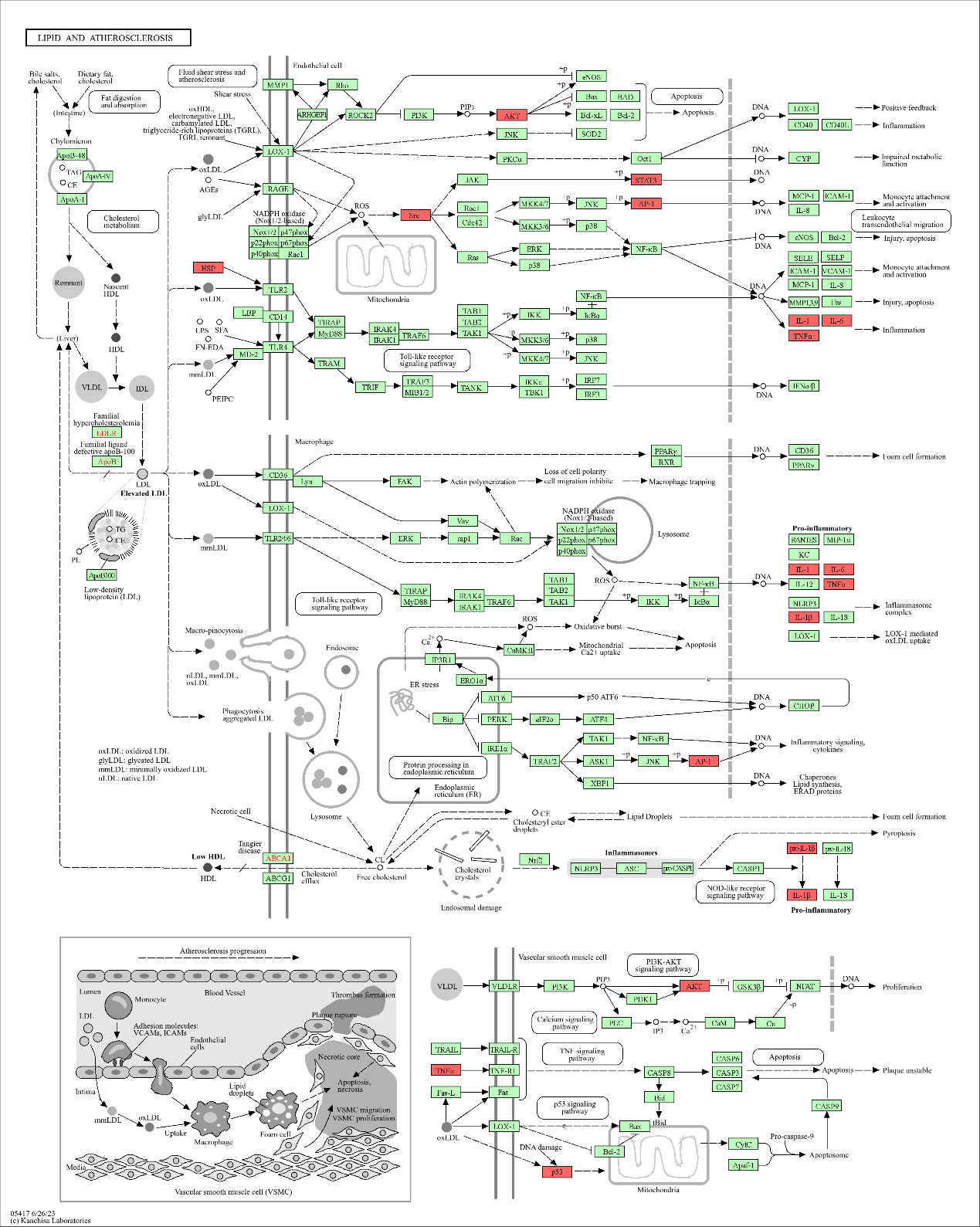


Supplemental Figure 3


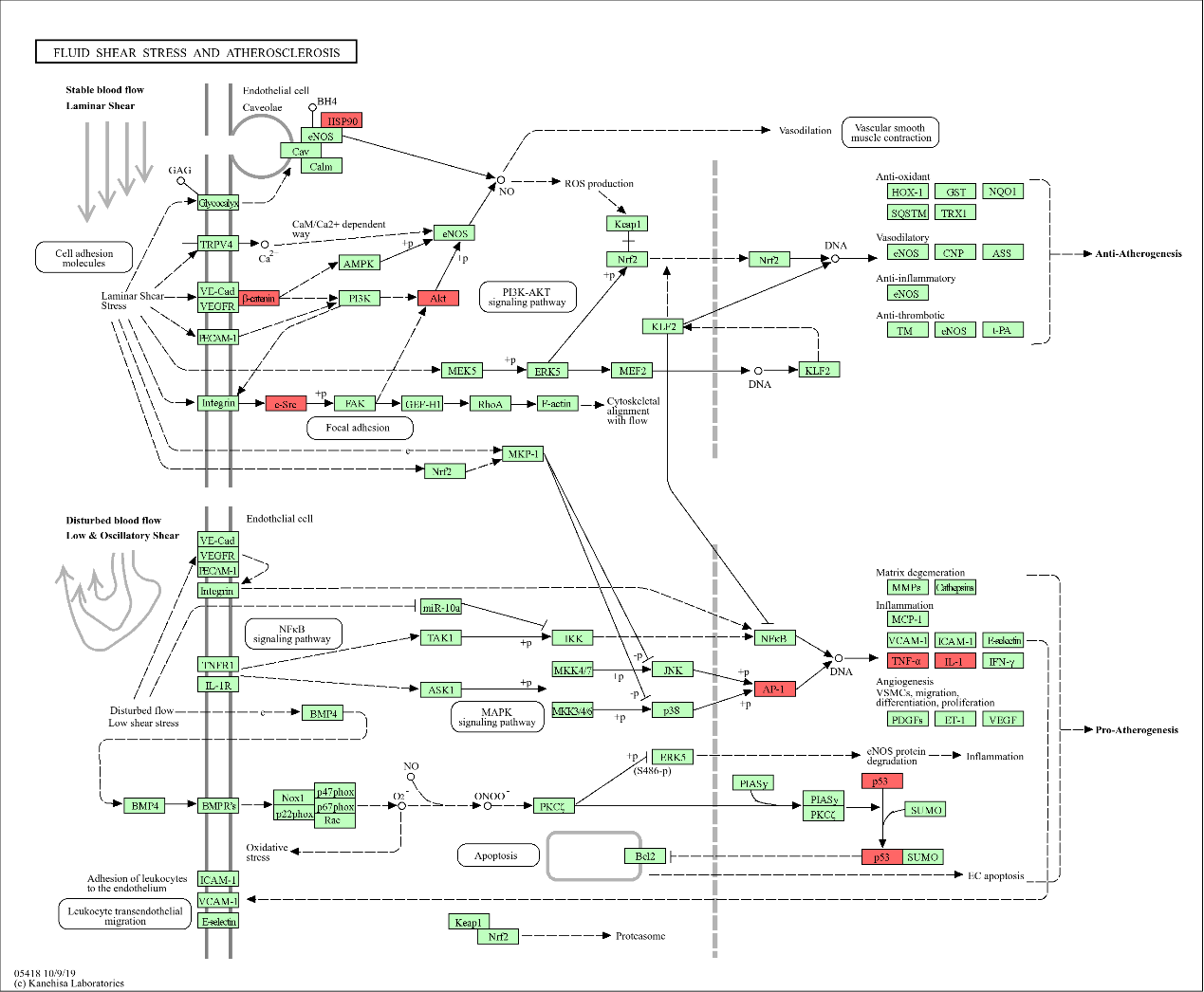

Supplement: Supplementary file 1 [file medi-103-e40073-s001.docx]
